# Spatial modeling of the population dynamics of *Anopheles* mosquitoes in Madagascar

**DOI:** 10.1186/s12942-025-00424-8

**Published:** 2025-11-18

**Authors:** Hobiniaina Anthonio Rakotoarison, Thiery Nirina Nepomichene, Hélène Guis, Romain Girod, Solofoarisoa Rakotoniaina, Fanjasoa Rakotomanana, Annelise Tran

**Affiliations:** 1 Epidemiology and Clinical Research Unit, Institut Pasteur de Madagascar, Antananarivo, Madagascar; 2 CIRAD, UMR TETIS, Montpellier, France; 3 TETIS, Univ Montpellier, AgroParisTech, CIRAD, CNRS, INRAE, Montpellier, France; 4 LGET, IOGA, Université d’Antananarivo, Antananarivo, Madagascar; 5 Medical Entomology Unit, Institut Pasteur de Madagascar, Antananarivo, Madagascar; 6 CIRAD, UMR ASTRE, Antananarivo, Madagascar; 7 ASTRE, Univ Montpellier, CIRAD, INRAE, Montpellier, France; 8 Epidemiology and Public Health Unit, Institut Pasteur du Cambodge, Phnom Penh, Cambodia; 9 CIRAD, UMR ASTRE, Montpellier, France

**Keywords:** Agricultural calendar integration, *Anopheles* population dynamics, Madagascar, Mechanistic model, Spatiotemporal simulation

## Abstract

**Background:**

Malaria, whose parasites are transmitted by *Anopheles* mosquitoes, remains a major public health burden in Madagascar despite the control measures led by the National Malaria Control Program. Understanding the population dynamics of *Anopheles* mosquitoes is therefore essential to optimize malaria surveillance and control. This study aimed to develop a model incorporating environmental, climatic and agricultural determinants of *Anopheles* abundance to predict their spatiotemporal distribution.

**Methods:**

We developed a model of spatiotemporal dynamics for four *Anopheles* species, vectors of malaria parasite in Madagascar: *Anopheles arabiensis*, *Anopheles coustani*, *Anopheles funestus* and *Anopheles gambiae*. This model was based on the life cycle of *Anopheles* and accounted for both the aquatic and aerial phases of their development. It used a system of differential equations to estimate the number of *Anopheles* mosquitoes at each stage of development. The Ocelet language, dedicated to the modeling of spatial dynamics, was used to produce simulations based on climate and environmental data. The model explicitly integrates the agricultural calendar to adjust the environmental carrying capacity of larval habitats. Model outputs were validated with entomological data collected in Vohimasy (Farafangana districts, 2014–2017).

**Results:**

24 simulation outputs, from three *Anopheles* species and eight sites, were obtained and the validation revealed a significant correlation between field observations and model predictions: the correlation coefficients obtained ranged from 0.70 to 0.76. The predicted abundance of host-seeking *Anopheles* varied seasonally influenced by precipitation, temperature and environmental carrying capacity. The model exhibited robustness across sites with diverse climates and accurately reproduced interannual dynamics. The integration of the agricultural calendar significantly reduced the overestimation of the density of host-seeking adult females.

**Conclusion:**

The developed *Anopheles* dynamics model provides a valuable tool for predicting mosquito abundance and distribution over time and space. It correctly predicted the abundance at villages with contrasting climates and reproduced interannual dynamics well. A distinctive aspect of this work lies in the explicit integration of seasonal agricultural practices into the estimation of larval habitat availability. This allows for a more accurate and transferable modeling of *Anopheles* population dynamics.

**Supplementary Information:**

The online version contains supplementary material available at 10.1186/s12942-025-00424-8.

## Background

Mosquitoes are among the common biting insects, and they are also the main vectors of pathogens such as viruses and parasites that can induce diseases such as dengue, Zika, chikungunya, filariasis, and malaria. The latter is the most common parasitic infection and continues to kill hundreds of thousands of people each year [1]. Malaria affects approximately 85 countries, particularly in tropical areas. In 2022, globally, the World Health Organization (WHO) estimated there were 249 million cases of malaria. Africa alone accounts for 92% of malaria cases [2].

In Madagascar, malaria continues to pose a significant public health challenge. Following a period of stability between 2018 and 2019, characterized by an incidence rate of 36.9‰, a notable increase was observed in 2020, with the incidence rising to 70.6‰, before reaching its peak in 2021 at 81.3‰ [3]. This resurgence has had a particularly pronounced impact on children aged 0 to 15 years [3]. Malaria transmission in Madagascar is heterogeneous and characterized by two epidemiological profiles [4]. In the Highlands and the subdesert South, malaria is unstable and is characterized by low transmission. In contrast, it is stable and marked by high transmission in the coastal regions. Despite the preventive and control measures implemented by the Ministry of Public Health, such as the indoor residual spraying, the distribution of long-lasting insecticidal nets, and intermittent preventive treatment for pregnant women, malaria persists in the country and remains a major public health problem.

*Plasmodium*, the pathogen responsible for malaria, is transmitted by certain species of mosquitoes of the genus *Anopheles*. Thus, malaria transmission is strongly influenced by the population dynamics of *Anopheles* [5, 6], whose aquatic stages (eggs, larvae, and pupae) as well as the aerial adult stage are affected by environmental and climatic conditions [5–7]. *Anopheles*, like all mosquitoes, are very sensitive to the temperature of the environment in which they live because they are poikilothermic, meaning that their body temperature is not stable and is highly dependent on the outside temperature [8]. Temperature also strongly impacts the length of development of the various stages of the mosquito life cycle by modulating their metabolism, which accelerates or slows down the transition between stages [9]. It also influences the development of pathogens in female *Anopheles*, particularly Plasmodium, by affecting the rates of maturation of parasitic forms, which conditions the duration of extrinsic incubation [5, 10]. Precipitation can greatly affect the availability of breeding sites. Environmental and climatic factors therefore strongly influence the distribution and spatiotemporal dynamics of malaria [11–14]. Global and climatic changes are altering landscape structures and disrupting the population dynamics of *Anopheles* in various ways, by altering their habitats, modifying breeding conditions, and influencing their life cycle, which directly impacts the emergence and spread of malaria [15]. For example, rising temperatures accelerate the mosquito life cycle and favor parasite development at moderate temperatures. Changes in precipitation patterns can increase breeding sites or, conversely, reduce them during drought periods. Deforestation, by creating temporary pools, and urbanization, by increasing stagnant water areas, also generate new habitats conducive to vectors. Finally, extreme weather events, such as floods, contribute to the emergence of new transmission hotspots [10, 15].

The dynamics of *Anopheles* mosquito populations are a key determinant of malaria transmission, influencing its intensity, duration, and seasonality [16]. These dynamics affect both the life cycle of the vectors and their competence in transmitting Plasmodium [9, 10]. Understanding these dynamics helps to better anticipate high-risk periods and areas, tailor control strategies, and optimize targeted interventions. Modeling these dynamics is a valuable tool for improving surveillance and enhancing the effectiveness of public health actions [17].

Vector control is one of the main measures to prevent malaria transmission in Madagascar [18]. The ability to predict *Anopheles* dynamics becomes essential to help public heath actors in malaria control. For example, one study showed that climatic factors strongly influence the dynamics of *Anopheles arabiensis* populations, which is crucial for assessing the effectiveness of control strategies [19]. Another model demonstrated that vector control interventions, such as insecticide-treated bed nets, affect the dynamics of *An. gambiae* populations, highlighting the importance of density-dependent regulation [20]. A further study integrated both abiotic and biotic factors to predict population dynamics and refine malaria control strategies [21]. In this context, modeling plays an important role, as it allows to describe the biology of *Anopheles*, and can act as a decision support tool for *Anopheles* surveillance and control. Several studies have resulted in the development of mosquito population dynamics models while highlighting the importance of climate and environment [19, 22–25]. Cailly et al. [22] developed a generic climate-based mosquito population model to evaluate vector control strategies, and found that climatic conditions strongly influence mosquito abundance, requiring control strategies adapted to these variations. In 2016, Abiodun et al. [19] investigated the influence of climatic factors on the temporal dynamics of *An. arabiensis* in the KwaZulu-Natal Province, South Africa, and found that these factors significantly affect the reproduction, maturation, and survival of this population. The generic mosquito population model [22] was applied in different geographical contexts and for different mosquito species, such as *Aedes albopictus* in Reunion Island [23, 25], and Rift Valley fever mosquito vectors in Senegal [24] and Botswana [26]. To our knowledge, this study is the first to explicitly incorporate local agricultural seasonality, particularly rice cultivation calendars, into a mechanistic framework for the dynamic estimation of the environmental carrying capacity of Anopheles larval habitats, and subsequently, the population dynamics of *Anopheles*. This integration formalizes a factor hitherto not accounted for in mechanistic mosquito population models, while providing a more accurate representation of the temporal availability of breeding habitats beyond climate-only approaches. Furthermore, our study simultaneously considers four *Anopheles* species that transmit malaria in Madagascar (*An. arabiensis*,* An. coustani*,* An. funestus* and *An. gambiae*). Some biological parameters were species-specific, while others, due to limited data or assumed similarities, were shared across species. This multi-species approach, which remains uncommon in mechanistic models, offers an improved representation of local vector dynamics without introducing excessive model complexity.

The objective of this study was to understand and predict the dynamics of *Anopheles* vectors of malaria in Madagascar by adapting the generic model developed by Cailly et al. [22] to the Malagasy context. This model accounted for both the temporal and spatial dimensions of *Anopheles* abundance.

## Methods

### Study areas

**Fig. 1:**
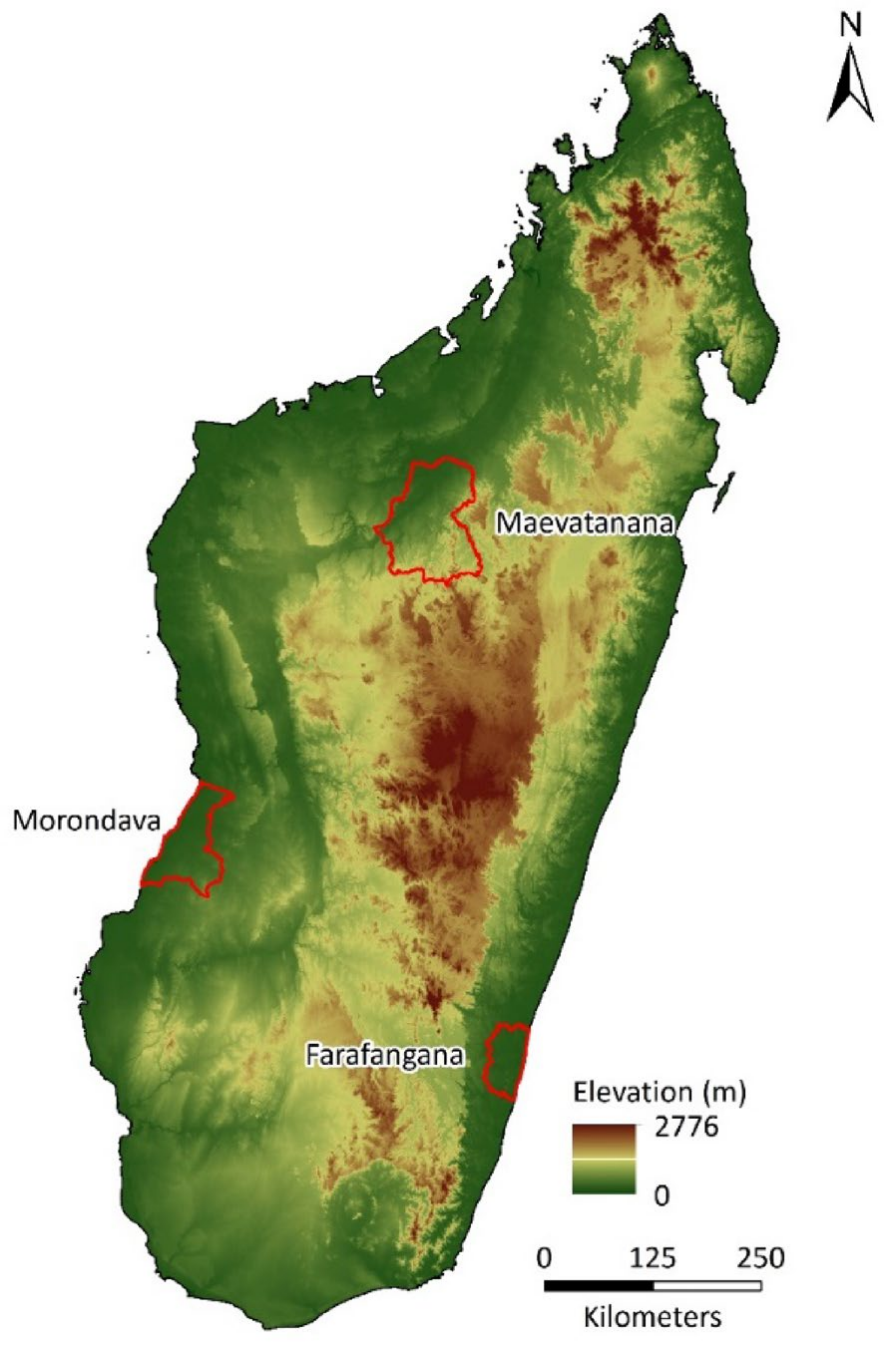
Location of the three study areas

Our study areas were three districts of Madagascar with different environments and climates: Farafangana District, Maevatanana District and Morondava District (Fig. 1).

Farafangana district, in the Atsimo Atsinanana region, is located in the southeastern part of Madagascar. Its altitude varies from 0 to 172 m. It is characterized by a hot and humid tropical climate. Although rainfall persists almost year-round, a less humid season is observed from April to August when drizzle replaces the heavy thunderstorms of November to March. This region is known for its high malaria incidence. The average annual rainfall in Farafangana District can reach a value of more than 2,500 mm [27]. The average temperature ranges from 15 °C in the dry season to 29 °C in the hot season [27].

Belonging to the Betsiboka region, Maevatanana District is located in the northwestern margins of the Central Highlands of Madagascar. It reaches an altitude of 1,479 m. The climate is tropical with an alternating dry season (April to November) and wet season (December to March). The average rainfall varies from 929 mm to 1,519 mm per year, while the average annual temperature is 21 °C. Although the region experiences a dry season, malaria transmission remains a concern due to seasonal variations in mosquito breeding, particularly in wetland areas.

The district of Morondava is located in western coast of Madagascar in the Menabe region. It is characterized by gently sloping land at an altitude between 0 m and 484 m. The average annual temperature is 19 °C, while the average annual rainfall is 570 mm. Despite its more arid conditions, Morondava faces significant malaria transmission due to the presence of wetlands and rivers, which create favorable breeding grounds for mosquitoes, particularly during the wet season.

## Data used

### Climatic data

Temperature and precipitation play a very important role in the life cycle of *Anopheles* [7].

The temperature and precipitation data used in this study were obtained from the International Research Institute (IRI) library (http://iridl.ldeo.columbia.edu/). These are meteorological estimates derived from remote sensing data from 2013 to 2017. Temperature data are provided in eight-day increments with a spatial resolution of 1 km, while precipitation data are available by decade with a spatial resolution of 12 km.

## Environmental data

The environment plays an important role in the life cycle of mosquitoes. The development of *Anopheles* is highly dependent on the land use, particularly wetlands that are potential breeding sites [28–30].

Landsat 8 satellite images were used to produce land use maps of the three study areas while focusing on wetlands representing potential breeding sites. These satellite images were downloaded from the United States Geological Survey (USGS) Earth Explorer website (https://earthexplorer.usgs.gov/). Wetlands (agricultural crops, water bodies, rivers and rice fields), which are potential breeding sites, were highlighted using supervised pixel-based classification. These land use types correspond to larval breeding sites of different *Anopheles* species. For rivers and water bodies, a buffer zone of two meters on each side of the edges of these two types of land use was set up and considered hatching sites. We assumed that small hive sites, not detected by satellite imagery, were already accounted for in the wetlands.

## Description of the model

**Fig. 2:**
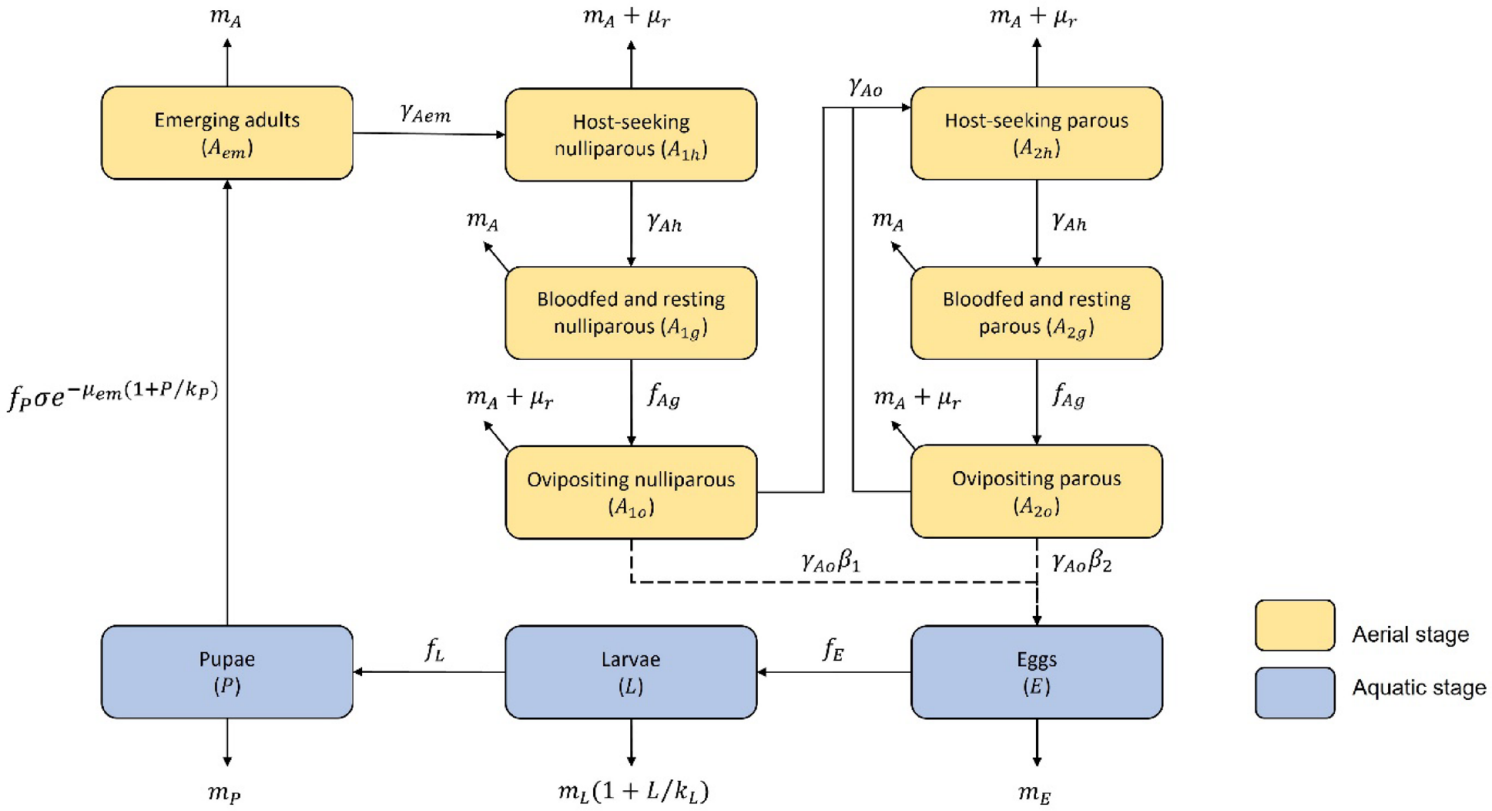
Compartment model representing the mosquito population dynamic

We adapted the generic mosquito dynamics model developed by Cailly et al. [22] to *Anopheles*. The initial model is a mechanistic model based on the life cycle process of mosquitoes with an aquatic phase (eggs, larvae and nymphs) and an aerial phase (adults). In this model, only female mosquitoes are considered. After emergence, the females mate and then take blood meals and lay their eggs, and the cycle starts again. The mosquito population is divided into ten compartments corresponding to the different developmental stage of mosquitoes (Fig. 2).

First, the eggs (*E*) become larvae (*L*) which then become pupae (*P*). After emergence, the emerging females (*Aem*) are considered nulliparous (i.e., females that have never laid eggs) in search of a host (*A1h*), i.e., in search of a blood meal, then engorged (*A1g*), and then in search for an oviposition site (*A1o*). After having laid eggs once, they become parous females in search of a host (*A2h*), then engorged (*A2g*) and finally in search for an oviposition site (*A2o*) (Fig. 2). Between each compartment, the mosquitoes move from one stage to another or die. To estimate the number of individuals for each developmental stage, a system of differential equations (Additional file 1) was used [22].

### Parameters and functions of the model

Transitions from one stage of *Anopheles* development to another, as well as mortality rates, are governed by parameters that do not vary in time or space (noted with Greek letters in Fig. 2) or functions (noted with Latin letters in Fig. 2) that depend on rainfall, temperature or environmental carrying capacity (i.e., the availability of breeding sites in the environment). The parameters used in the model are listed in Table 1, whereas Table 2 shows the functions used. They were defined from literature reviews and experiments. Some parameters were specific to each *Anopheles* species, while others were generalized to the *Anopheles* genus when species information was not available. The parameters that significantly influence variations in the peak of adult abundance, such as the standard environment carrying capacity, the mortality rate at emergence, the sex-ratio, and the number of eggs laid by parous females, were identified as particularly sensitive during the model’s sensitivity analysis (see [22] for details). To ensure robust results, it is essential to measure these variables with optimal precision, as they play a key role in the dynamics of the modeled populations.

**Table 1:** Parameters used in the model

Notation	Definition	*An. arabiensis*	*An. coustani*	*An. funestus*	*An. gambiae*	Reference
$\:{\beta\:}_{1}$	Number of eggs laid by nulliparous female/oviposition	48	72	68	71	[40, 45, 47]
$\:{\beta\:}_{2}$	Number of eggs laid by parous female/oviposition	64	96	108	105	[40, 45, 47]
$\:\sigma\:$	Sex-ratio at emergence	0.82	0.72	0.72	0.61	[41, 42, 46, 49]
$\:{\gamma\:}_{Aem}$	Development rate of emerging adults (day^−1^)	0.8	0.8	0.8	0.8	[43]
$\:{\gamma\:}_{Ah}$	Transition rate from host-seeking to engorged adults (day^−1^)	2	2	2	2	[48]
$\:{\gamma\:}_{Ao}$	Transition rate from ovipositing to host-seeking adults (day^−1^)	2	2	2	2	[48]
$\:{\mu\:}_{E}$	egg mortality rate (day^−1^)	0.1	0.1	0.1	0.1	[48]
$\:{\mu\:}_{L}$	Larvae mortality rate (day^−1^)	0.08	0.08	0.08	0.08	[48]
$\:{\mu\:}_{P}$	Pupae mortality rate (day^−1^)	0.01	0.01	0.01	0.01	[48]
$\:{\mu\:}_{em}$	Mortality rate during emergence (day^−1^)	0.1	0.1	0.1	0.1	[22]
$\:{\mu\:}_{r}$	Adult mortality rate related to seeking behaviour (day^−1^)	0.08	0.08	0.08	0.08	[22]
$\:{T}_{E}$	Minimal temperature needed for egg development (°C)	13.1	12.9	12.7	12.9	[44]
$\:{T}{D}{D}{E}$	Total number of degree-days necessary for egg development (°C)	25.4	26.6	35.6	26.6	[44, 48]
$\:{T}_{Ag}$	Minimal temperature needed for egg maturation (°C)	9.9	9.9	9.9	9.9	[48]
$\:{TDD}_{Ag}$	Total number of degree-days necessary for egg maturation (°C)	36.5	36.5	36.5	36.5	[48]
$\:K$	Standard carrying capacity of aquatic stages (Larvae/m²)	1914	1914	1914	1914	*

* To the best of our knowledge

**Table 2:** Functions of the model

Notation	Definition	Expression	Reference
$\:{f}_{E}$	Transition function from egg to larva	$\:\frac{T\left(t\right)-{T}_{E}}{{TDD}_{E}}$	[22]
$\:{f}_{L}$	Transition function from larva to pupa	$\:\frac{{f}_{P}}{4}$	[22]
$\:{f}_{P}$	Transition function from pupa to emerging adult	$\:0.021*\frac{{e}^{0.162\left(T\right(t)-10)}-{e}^{0.162*25-35+T\left(t\right)}}{5.007}$	[22]
$\:{f}_{Ag}$	Transition function from engorged adult to oviposition site-seeking adult	$\:\frac{T\left(t\right)-{T}_{Ag}}{{TDD}_{Ag}}$	[22]
$\:{m}_{L}$	Mortality function for larva	$\:{e}^{\frac{-T\left(t\right)}{2}}+{\mu\:}_{L}$	[31]
$\:{m}_{P}$	Mortality function for pupa	$\:{e}^{\frac{-T\left(t\right)}{2}}+{\mu\:}_{P}$	[31]
$\:{m}_{A}$	Mortality function for adult	$\:0.1-0.00667T\left(t\right)+0.000148T^2\left(t\right)$	[31]
$\:{k}_{X}$	Environment carrying capacity for aquatic stage	Equation 1	*

* To the best of our knowledge

Larval and pupal dynamics are dependent on the availability and the suitability of breeding sites, expressed as the environmental carrying capacity. The environmental carrying capacity function (Eq. 1) depends on precipitation. Indeed, precipitation influences the availability of breeding sites [19].

1 $$\:{k}_{X}={K}_{fix}\left(t\right)+\left({K}_{var}\left(t\right)*{P}_{norm}\left(t\right)\right),\:\:\:\:\:\:\:\:\:X\in\:\left\{L,\:P\right\}$$

where is the precipitation-independent environmental carrying capacity; is the precipitation-dependent environmental loading capacity; and is the weekly precipitation normalized between 0 and 1. The variable *Pnorm* to represent weekly precipitation normalized between 0 and 1. This normalization was performed at the local scale (by district), over the entire study period (2013–2017), using the observed minimum and maximum weekly values. When precipitation is zero, *Pnorm* = 0, reflecting the absence of temporary breeding sites. When precipitation reaches its local maximum, *Pnorm* = 1, indicating the highest possible contribution of rainfall-dependent breeding sites. This approach allows for a flexible and biologically realistic modulation of the environmental carrying capacity related to rainfall, while ensuring comparability across sites.

For each study site the fixed and variable load capacities were estimated for each month following Eqs. 2 and 3:

2 $$\:{K}_{fix}\left(t\right)=\sum\:_{gites=1}^{4}{{S}_{breeding\:sites}*K}_{{fix}_{breeding\:sites}}\left(t\right)$$

3 $$\:{K}_{var}=\sum\:_{gites=1}^{4}{{S}_{breeding\:sites}*K}_{{var}_{breeding\:sites}}\left(t\right)$$

where *breeding s* is the surface area of the larval breeding sites and * breeding s* and *breeding s* are the fixed and variable carrying capacities of each larval site (river, agricultural crop, water body and rice field), respectively.

Considering that *Anopheles* larval sites in Madagascar are mainly dependent on rainfall, except for rice fields when they are in water (August and September), we estimated the fixed and variable carrying capacities for each type of larval site as follows:

4 $$\begin{aligned} & \:{K}_{{fix}_{breeding\:sites}}\left(t\right) \\ & \quad =\left\{\begin{array}{ll} K*0.1 & \text{for breeding sites} \\ & \quad \in \{\text{river, agricultural crop,water body}\}, \forall t \\ K*0.1 & \text{for breeding sites=rice field} \,and\, t\\ & \quad \notin\{\text{August, September}\} \\ K & \text{for breeding sites}=\text{rice field} \,and \,t \\ & \quad \in\{\text{August, September}\} \end{array}\right. \end{aligned}$$

5 $$\begin{aligned} & \:{K}_{{var}_{breeding\:sites}}\left(t\right) \\ & \quad =\left\{\begin{array}{ll} K*0.9 & for \,breeding \,sites \\ & \quad \in \{\text{river, agricultural crop,water body}\}, \forall t \\ K*0.9 & for \,breeding\, sites=rice \,field \,and\, t\\ & \quad \notin\{\text{August, September}\} \\ 0 & for \,breeding \,sites=rice \,field \,and \,t \\ & \quad \in\{\text{August, September}\} \end{array}\right. \end{aligned}$$

## Simulation

The simulation of the model was performed with the language “Ocelet”, which is a computer language specialized in the simulation of spatial dynamics, developed by CIRAD [32], for the period from 2013 to 2017. The first year was used as an initialization year, and 10^7^ eggs were initially considered to start the simulation. In the end, the outputs of this model were estimates for each species, at each time step (every week in this study), of the number of individuals in each of the *Anopheles* developmental stages (*E, L, P, Aem, A1h, A1g, A1o, A2h, A2g, A2o*) in each commune of the three districts considered.

To assess the impact of the rice-cultivation calendar on the larval carrying capacity ($\:{k}_{X}$), two scenarios were compared over the period 2014–2017: (i) a model without consideration of the agricultural calendar and (ii) a model incorporating the agricultural calendar via the function $\:{K}_{var}$ described in Eqs. 1–5. Both scenarios were then simulated to quantify the influence of rice seasonality on the dynamics of *Anopheles* population.

## Validation

The model was validated by comparing the predicted host-seeking density of *Anopheles* (*Aℎ = A1ℎ + A2ℎ*) with entomological data provided by the Medical Entomology Unit of the Institut Pasteur of Madagascar. These include the number of mosquitoes collected every two months between 2014 and 2017 at Vohimasy, between 2014 and 2016 at Mahasoa, and in 2017 at Ambahibe, in the Farafangana district. Initially, Mahasoa had been selected as the second site, alongside Vohimasy, for entomological collections throughout the 2014–2017 period. However, due to security issues, the Mahasoa site was abandoned in 2017, and collections were transferred to Ambahibe. For this study, we focused exclusively on the main *Anopheles* species acting as malaria vectors in the study areas: *An. arabiensis*, *An. coustani*, *An. funestus*, and *An. gambiae* [33–35]. Among these species, we considered the three most aggressive species in each study area. The Spearman correlation coefficient was computed for each *Anopheles* species to evaluate the congruence between predicted and observed densities.

## Results

### Temporal dynamics

**Fig. 3:**
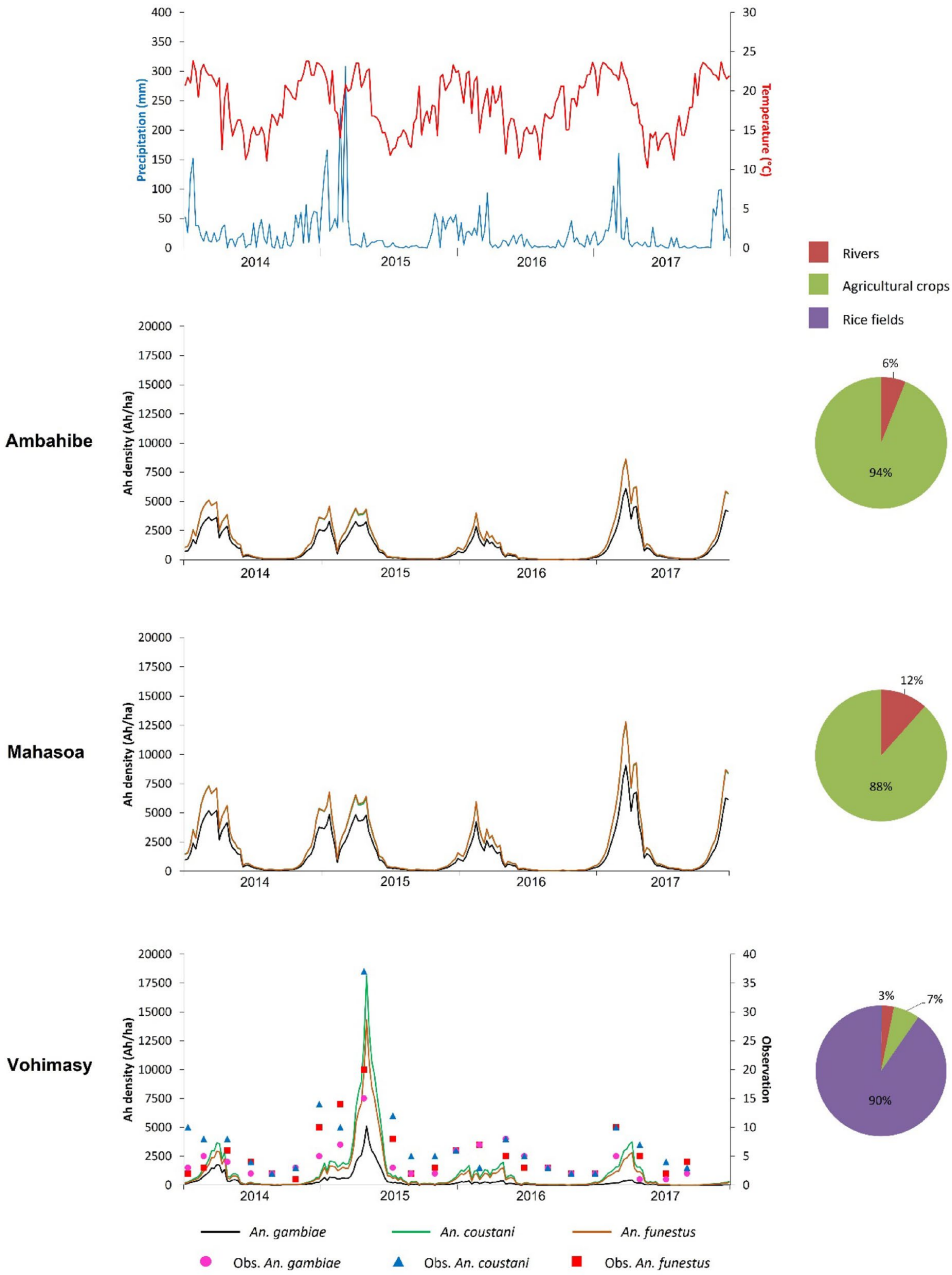
Population dynamics of host-seeking *Anopheles* at the three sites in Farafangana District. *The curves correspond to predicted densities*,* the dots represent field observations*,* and the pie-charts represent the breeding sites*

**Fig. 4:**
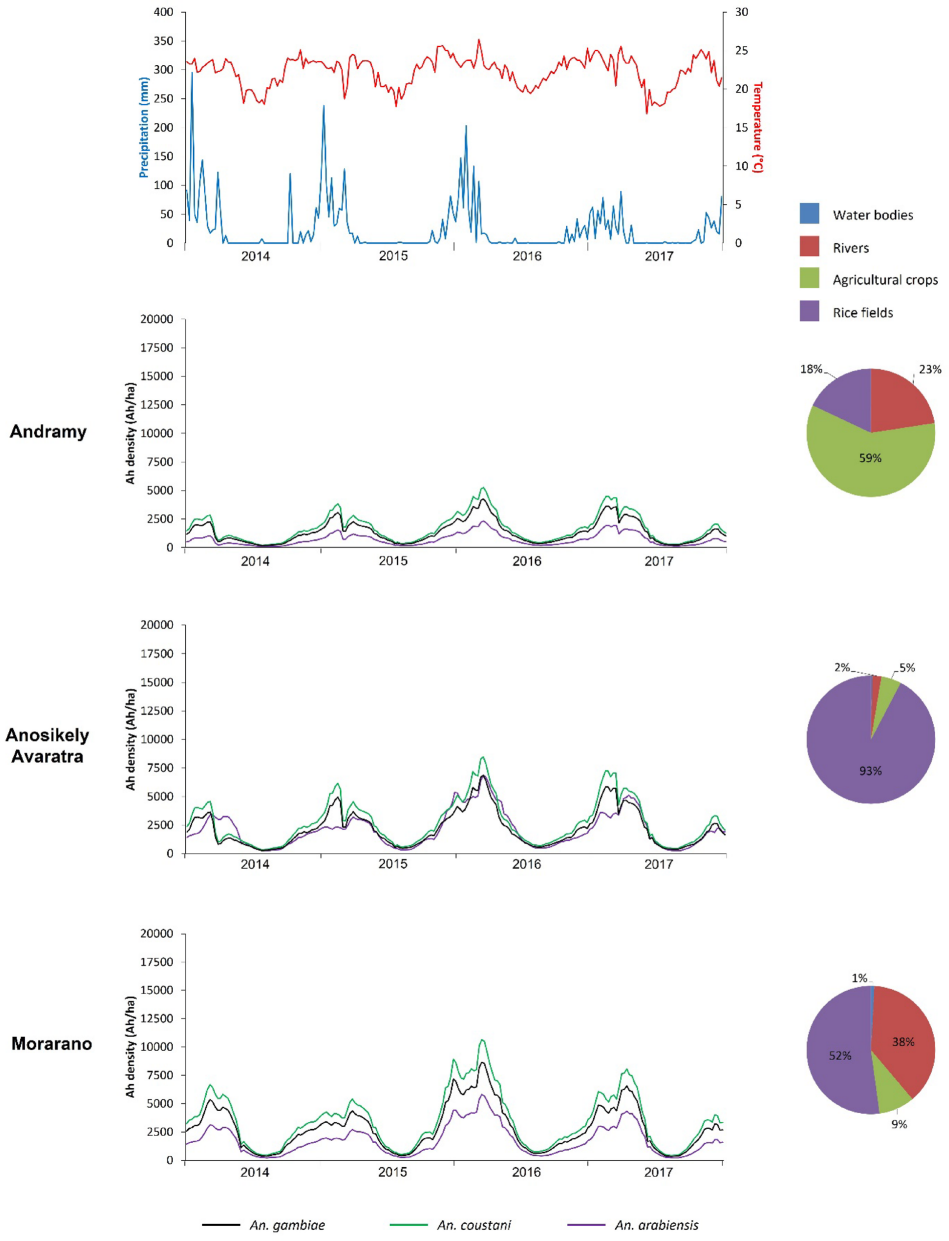
Population dynamics of host-seeking *Anopheles* at the three sites in Maevatanana District

**Fig. 5:**
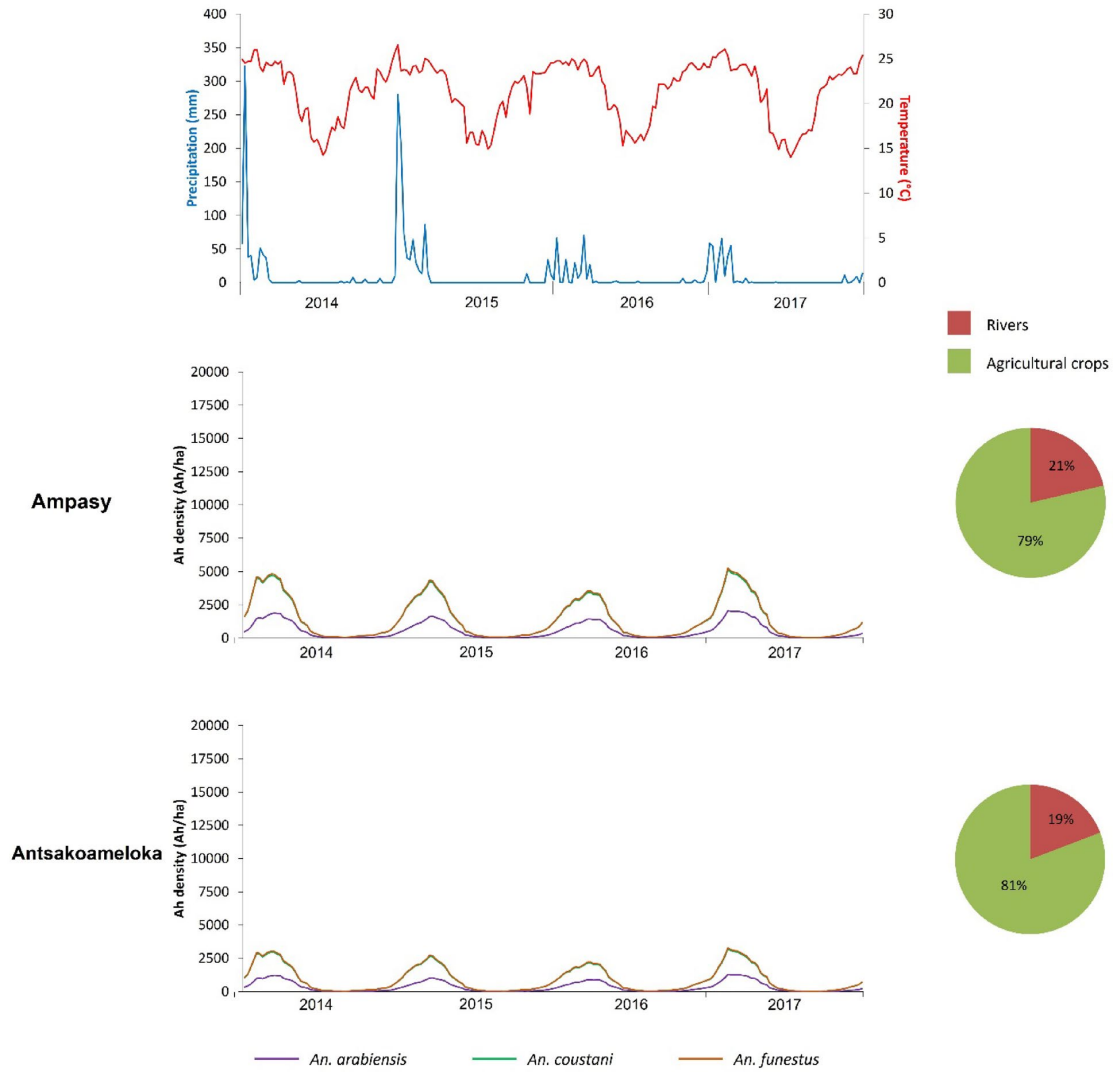
Population dynamics of host-seeking *Anopheles* at the two sites of Morondava District

A total of 24 simulation outputs was generated, corresponding to the three modeled *Anopheles* species across the eight study sites. The results revealed temporal dynamics in the predicted abundance of host-seeking anopheles’ mosquitoes (Figs. 3, 4 and 5).

In the three study sites of the Farafangana district (Ambahibe, Mahasoa, and Vohimasy), *Anopheles* densities exhibited recurrent annual peaks during the hot and rainy season (Fig. 3). These peaks were particularly pronounced in 2017 at Ambahibe and Mahasoa, whereas previous years showed more moderate densities. At Vohimasy, a sharp increase in mosquito density was observed in 2015. *An. funestus* displayed the highest densities, followed by *An. coustani*; these two species were dominant in Ambahibe and Mahasoa, where their abundance levels were nearly equivalent. In contrast, *An. gambiae* remained relatively scarce, particularly at Vohimasy. In the first two sites, annual peaks occurred each year, with a marked maximum in 2017 at Ambahibe, while earlier years showed lower values. At Vohimasy, densities also varied between years, with a particularly strong increase in 2015. This site is characterized by a high prevalence of rice fields (90% of larval habitats), in contrast to the other two sites, which were primarily dominated by agricultural crops (> 88%), with no rice fields present. Finally, precipitation and temperature data exhibited seasonal variability, with precipitation maxima generally coinciding with peaks in *Anopheles* density.

The sites of Andramy, Anosikely Avaratra, and Morarano (Maevatanana district) exhibited regular seasonal dynamics from 2014 to 2017, marked by density increases during the rainy periods (Fig. 4). No extreme fluctuations were recorded during this timeframe. *An. coustani* was the dominant species across all three sites, followed by *An. gambiae* and then *An. arabiensis*. This ranking remained broadly stable over the years. Larval habitat profiles differed among sites: rice fields predominated in Anosikely Avaratra and Morarano (93% and 52%, respectively), with Morarano also exhibiting a high proportion of riverine areas (38%), whereas agricultural crops were predominant in Andramy (59%). All three habitat types were present at each site, along with small proportions of water bodies in Morarano (1%) and Anosikely Avaratra (2%). Climatic data showed a marked seasonality in precipitation, synchronized with density peaks. Temperatures, on the other hand, exhibited less pronounced variations.

In the Morondava district (Ampasy and Antsakoameloka sites), the observed dynamics followed a similarly regular annual pattern, with consistent density peaks during the hot and humid season from 2014 to 2017 (Fig. 5). No exceptional variations were observed over the period. The mosquito populations were mainly composed of *An. funestus* and *An. coustani*, with comparable densities. *An. arabiensis* was present at lower densities. This species composition remained relatively stable from year to year. In terms of larval habitats, agricultural crops clearly predominated, accounting for 79% in Ampasy and 81% in Antsakoameloka. Riverine areas complemented these habitats, representing 19% and 21%, respectively. Climatic conditions maintained a seasonal pattern: rainfall, although slightly variable from year to year, coincided with periods of high *Anopheles* density. Temperatures remained stable, with moderate fluctuations.

For the Vohimasy site in the Farafangana District, a comparison between simulation outputs and observed entomological field data from 2014 to 2017 showed that the model correctly predicted the dynamics of anopheles mosquito (*An. gambiae*,* An. coustani*,* An. funestus*). The predicted densities of host-seeking *Anopheles* fit well with those observed in the field and were well correlated, with correlation coefficients of 0.70 for *An. gambiae*, 0.76 for *An. coustani*, and 0.73 for *An. funestus*.

**Fig. 6:**
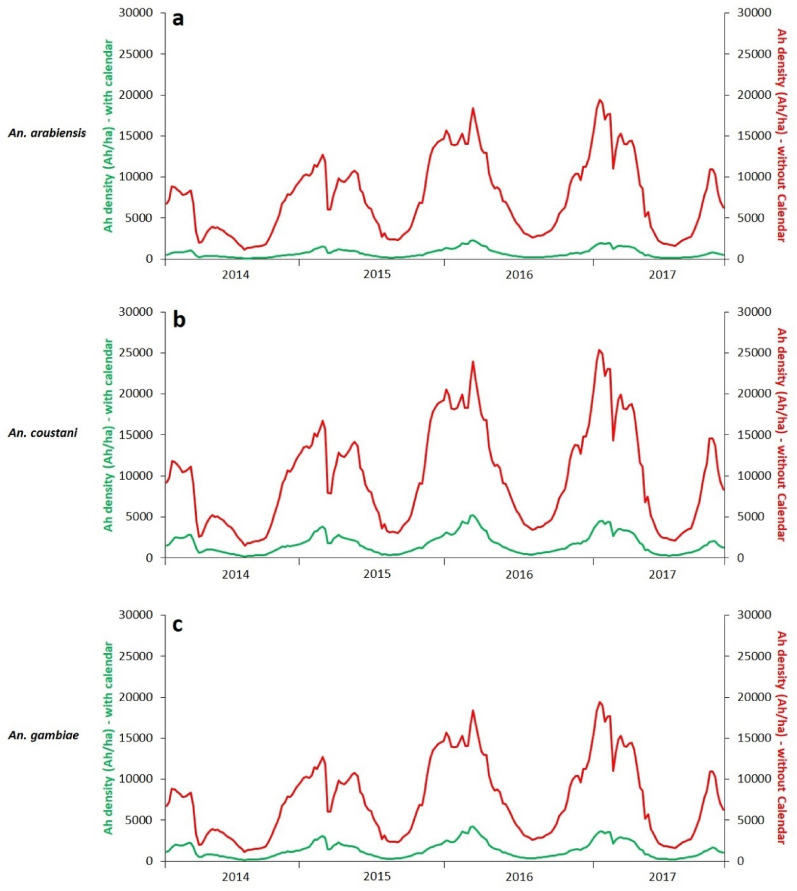
Comparison of simulations with and without the agricultural calendar in Andramy (Maevatanana). **a**: *An. arabiensis*, **b**: *An. coustani*, **c**: *An. gambiae*

In Andramy site, simulated densities of host-seeking *Anopheles* (Ah) were compared between a model without the agricultural calendar and one incorporating it (Fig. 6). For the three species *An. arabiensis*,* An. coustani*, and *An. gambiae*, simulations without the agricultural calendar consistently displayed markedly higher densities than those obtained with the agricultural calendar over the entire 2014–2017 period. The curves without the agricultural calendar reached maximum values between 19,000 Ah·ha⁻¹ and 25,000 Ah·ha⁻¹, whereas simulations with the agricultural calendar showed much lower levels, with a maximum peak of 4,890 Ah·ha⁻¹. This pronounced difference was observed repeatedly throughout the simulated seasonal cycles. Simulations from other study sites and species revealed the same general pattern (Additional file 2).

### Spatial dynamics

**Fig. 7:**
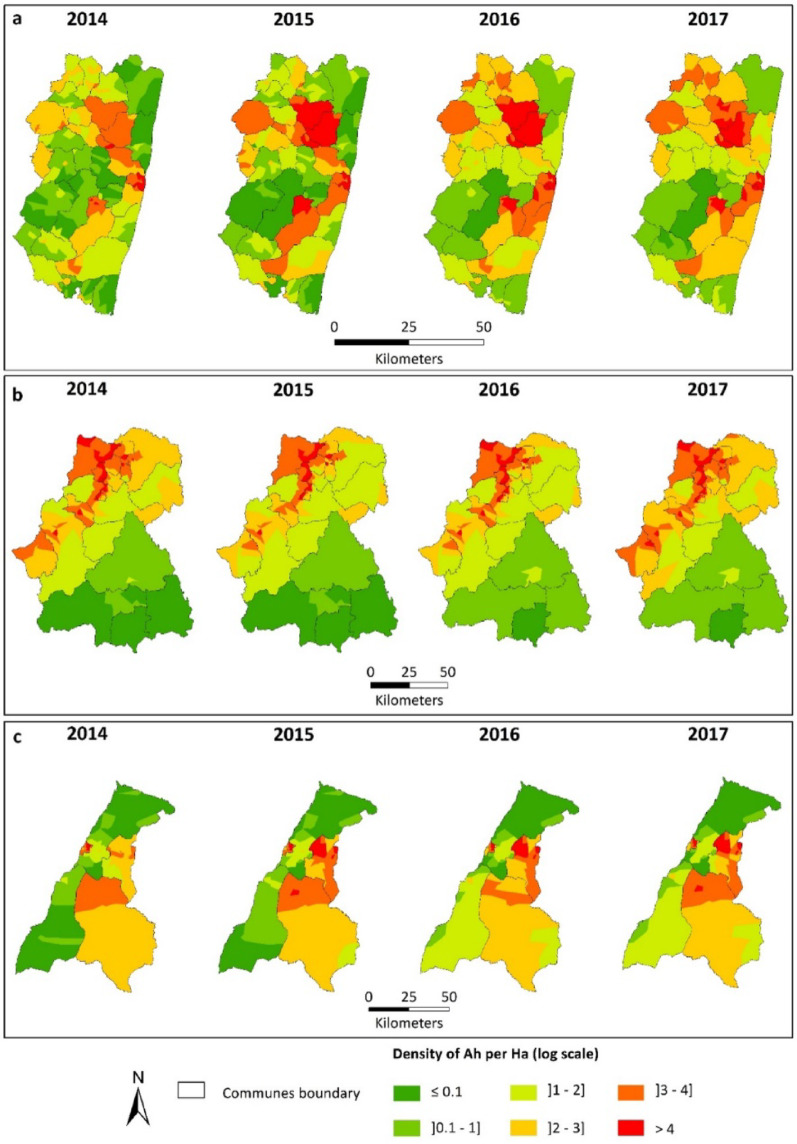
Maps of the annual densities of ℎ for 2014, 2015, 2016 and 2017. **a**: Farafangana district, **b**: Maevatanana district, **c**: Morondava district

Maps showing the spatial distribution of the annual mean density of host-seeking *Anopheles* mosquitoes for the years 2014 to 2017 were obtained, based on the model results. These maps cover the three study districts and reveal substantial spatial heterogeneity (Fig. 7).

In Farafangana, high-density areas in 2014 were mainly concentrated in the north-central region and parts of the eastern zone of the district. In contrast, the northeastern and central areas displayed predominantly low to very low densities, while the southern part exhibited low to moderate densities. In the following year, densities in the north-central zone increased from high to very high. A new high-density area also emerged in the south-central part of the district, contrasting with low to moderate densities observed elsewhere. In 2016, density levels increased across most communes. The year 2017 was characterized by a more balanced spatial distribution, with medium densities becoming more common and an overall attenuation of extreme values (Fig. 7a).

In Maevatanana, the maps also reveal a markedly heterogeneous distribution (Fig. 7b). In 2014, the northern half of the district concentrated most of the high to very high-density zones, while the southern half was dominated by very low to low densities. This configuration remained largely stable in 2015. In 2016, a one-level increase in density was observed in the southern part of the district, with values rising from very low to low, while little change was noted elsewhere. In 2017, overall density levels remained relatively stable, but spatial heterogeneity persisted.

In Morondava, the spatial distribution remained relatively stable across years, though disparities were still present (Fig. 7c). In 2014, the highest densities were observed in the central area and in some parts of the east and west. In contrast, the south and the remaining western areas showed low to moderate densities. This pattern remained mostly unchanged in 2015, except for a slight southward extension of low-density areas and the emergence of a more pronounced hotspot in the eastern region. In 2016, low-density zones continued to expand in the southeast, where very low densities shifted to low levels. The rest of the district exhibited a generally stable spatial pattern. In 2017, the distribution of host-seeking *Anopheles* densities was similar to that of the previous year, except in the central area, where densities increased from moderate to high levels.

## Discussion

This study aimed to improve the understanding and prediction of *Anopheles* population dynamics in Madagascar, by adapting a generic life-cycle-based mechanistic model to local ecological and agricultural contexts, shows that the explicit integration of agricultural cultivation significantly influences the dynamic estimation of larval habitat carrying capacity. To our knowledge, no previous model had explicitly included such an agro-environmental factor to assess the availability of larval habitats.

Comparative simulations (Fig. 6 and Additional file 2) indicate that incorporating the agricultural calendar markedly dampens the dynamics of host-seeking female *Anopheles* populations by reducing the overestimations observed in models based solely on climatic variables, with predicted densities frequently several times lower. This difference, consistently observed across simulated seasonal cycles, suggests that variations in the availability and quality of larval habitats driven by agricultural practices exert a major regulatory effect on adult populations. Thus, beyond climatic fluctuations alone, the integration of agro-environmental variables appears essential for more realistically reproducing vector dynamics and for improving the temporal relevance of control strategies, particularly in targeting periods with the highest potential effectiveness of interventions.

Although this application focuses on Madagascar, the methodology, based on integrating local agricultural practices into a mechanistic framework, can be generalized to other regions where agriculture, particularly rice cultivation or other forms of irrigated farming, strongly shapes the dynamics of aquatic habitats, such as parts of West Africa (e.g., the Senegal River Valley) or Southeast Asia (e.g., the Mekong Basin). The explicit integration of agricultural calendars into mechanistic models thus offers an innovative, transferable, and relevant methodological pathway for anticipating vector dynamics in diverse agricultural settings.

The results highlight two essential aspects of malaria vector dynamics in Madagascar: strong seasonality and marked spatial heterogeneity. These two characteristics point to distinct regulatory mechanisms in vector population dynamics. The observed seasonality reflects a clear response of *Anopheles* populations to annual climatic variations, particularly rainfall and temperature, which directly influence the availability of larval habitats and adult mosquito survival. However, this study refines the understanding of these dynamics by showing that periods of high abundance are relatively predictable (from November of year *n* to April of year *n* + 1, corresponding to the warm and wet season), which could ultimately allow better anticipation of transmission periods. The spatial contrasts observed, even between nearby localities, indicate that local-specific factors, beyond climate, influence vector distribution. This suggests that vector dynamics are not spatially uniform and that environmental, ecological, or human-specific conditions play a key role. This spatial heterogeneity underscores the importance of using localized approaches to understand and anticipate variations in transmission risk.

The seasonal and spatial dynamics of *Anopheles* populations observed in this study are consistent with findings from several previous mechanistic modeling studies, including Cailly et al. (2012) [22] and Ezanno et al. (2015) [36], which emphasized the key role of climatic factors in shaping vector densities. However, our approach stands out by explicitly incorporating the effects of local agricultural practices, especially the rice-growing calendar, into the modeling of larval habitats. This choice allowed for a more accurate representation of environmental carrying capacity fluctuations in relation to both climatic seasons and agricultural practices specific to Madagascar. Precipitation and agricultural calendars were used to dynamically adjust the availability of larval habitats, resulting in a more realistic depiction of the environment. By comparison, the models by Cailly et al. and Ezanno et al. used fixed carrying capacities that did not evolve with habitat changes. Abiodun et al. (2016) [19] introduced variability in carrying capacity, but without considering local agricultural features. Other mechanistic models, such as VECTRI [37], have highlighted the value of using diverse datasets to simulate malaria transmission at a regional scale. While our study does not explicitly model disease transmission, it follows a similar logic by incorporating local dynamics to better explain the spatial heterogeneity observed between sites, sometimes even at sub-district scales. More recent studies, such as Ngowo et al. (2022) [38] in Tanzania, using Bayesian models to explore climate change impacts, confirm the growing importance of spatially explicit and context-sensitive approaches to malaria vector research. Our results build on these efforts by offering a mechanistic formalism that is directly applicable to local contexts. Furthermore, statistical approaches based on machine learning, such as those used by Taconet et al. (2021) [39], have proven effective in linking remote sensing-derived environmental variables to vector density or biting indices. While these methods are useful for identifying broad-scale correlations, they generally do not allow for intervention scenario simulations. In this sense, they are complementary to mechanistic models, which can explicitly test hypotheses regarding control factors. The model presented here thus contributes to this complementarity by providing a mechanistic framework informed by local parameters, with a view toward future applications in targeted vector risk management.

The model developed in this study offers concrete perspectives for the spatiotemporal planning of vector control interventions. By identifying periods and locations of high vector density, it enables optimization of interventions such as indoor residual spraying, larviciding, or the distribution of insecticide-treated nets. Its integration into entomological surveillance or early warning systems could help shorten response times and more effectively target available resources. The model was applied here to the period 2014–2017 to calibrate and validate its ability to reproduce observed dynamics based on available entomological data. This validation step is essential before any operational deployment. In the short term, the model is designed for prospective use, with the objective of generating updated maps of vector density to support public health stakeholders in managing the risk of malaria transmission more effectively.

Very little information is available in the study areas on parameters that affect the life cycle of *Anopheles*. Laboratory experiments on these parameters, and particularly on the influence of climatic conditions on the survival and development of *Anopheles*, could help improve the population dynamics model. Indeed, most of the parameters and functions used in this study were drawn from literature on *Anopheles* species in regions other than Madagascar. Nepomichene et al. [40] estimated the average number of eggs laid per female for *An. coustani*,* An. funestus*,* and An. arabiensis* in Madagascar. Some parameters were estimated for the target species (*β* for *An. gambiae*, *σ*, *γAem*, *TE*, and *TDDE* for *An. arabiensis* and *An. funestus*) but in non-Malagasy settings [41–47]. Other parameters, such as *γAh*, *γAo*,* µE*,* µL*,* µP*,* µem*,* µr*,* TDDE* (*An. coustani*,* An. gambiae*), *TAg*, and *TDDAg*, have been estimated for other *Anopheles* species [22, 48].

In Madagascar, only 25 weather stations cover the entire island. This number is far too low for a territory of 587,041 km², and the risk of interpolation bias is therefore high. This scarcity of meteorological stations necessitates the use of remotely sensed meteorological estimates. However, most of the available remote sensing products offer relatively low spatial resolution, which limits their suitability for local-scale studies.

## Conclusion

This work constitutes, to our knowledge, the first study demonstrating the impact of formally integrating the agricultural calendar into a mechanistic model of Anopheles population dynamics. In addition to this agro-environmental integration, the model was specifically adapted to four malaria vector species present in Madagascar: *An. arabiensis*, *An. coustani*, *An. funestus*, and *An. gambiae*. This capacity to account for species diversity enhances the model’s usefulness in areas where multiple vectors contribute differently to malaria transmission dynamics, making it a valuable and easily transferable tool for anticipating vector dynamics in other malaria-endemic regions where agriculture shapes mosquito ecology.

The approach developed here can be transposed to other agricultural regions where production systems govern the availability of larval habitats. Extending beyond the Malagasy context, it provides a transferable methodological basis for modeling mosquito dynamics.

The results also highlight the importance of environmental and climatic factors in shaping *Anopheles* abundance. The outputs of the model, describing *Anopheles* abundance in space and time, can be directly used to test vector control strategies or as input data for malaria transmission risk models, which could then be validated using malaria incidence data. Such applications would help identify and implement the most effective, context-specific combinations of vector and malaria control measures.

## Supplementary Information

Supplementary Material 1

Supplementary Material 2

## Data Availability

No datasets were generated or analysed during the current study.

## References

[1] WHO. World malaria report 2022. Geneva, Switzerland: World Health Organization; 2022.

[2] WHO. World malaria report 2023. Geneva, Switzerland: World Health Organization; 2023.

[3] PNLP. Plan Stratégique National 2023–2027. Antananarivo. Madagascar: Ministère de la Santé Publique - Direction de Lutte contre les Maladies Transmissibles; 2023.

[4] Howes RE, Mioramalala SA, Ramiranirina B, Franchard T, Rakotorahalahy AJ, Bisanzio D, et al. Contemporary epidemiological overview of malaria in Madagascar: operational utility of reported routine case data for malaria control planning. Malar J. 2016;15:502. 27756389 10.1186/s12936-016-1556-3 PMC5070222

[5] Beck-Johnson LM, Nelson WA, Paaijmans KP, Read AF, Thomas MB, Bjørnstad ON. The effect of temperature on *Anopheles* mosquito population dynamics and the potential for malaria transmission. PLoS ONE. 2013;8:e79276. 24244467 10.1371/journal.pone.0079276 PMC3828393

[6] Beck-Johnson LM, Nelson WA, Paaijmans P, Read AF, Bjørnstad ON. Population dynamics and malaria risk. R Soc Open Sci. 2017;4:11. 10.1098/rsos.160969 PMC5383843 28405386

[7] Moller-Jacobs LL, Murdock CC, Thomas MB. Capacity of mosquitoes to transmit malaria depends on larval environment. 2014;12. 10.1186/s13071-014-0593-4 PMC4273441 25496502

[8] Bligh J, Johnson KG. Glossary of terms for thermal physiology. J Appl Physiol. 1973;35:21. 10.1152/jappl.1973.35.6.941 4765838

[9] Christiansen-Jucht C, Parham PE, Saddler A, Koella JC, Basáñez M-G. Temperature during larval development and adult maintenance influences the survival of *Anopheles gambiae* s.s. Parasit Vectors. 2014;7:489. 25367091 10.1186/s13071-014-0489-3 PMC4236470

[10] Paaijmans KP, Read AF, Thomas MB. Understanding the link between malaria risk and climate. Proc Natl Acad Sci U S A. 2009;106:13844–9. 19666598 10.1073/pnas.0903423106 PMC2720408

[11] Kelly-Hope LA, Hemingway J, McKenzie FE. Environmental factors associated with the malaria vectors *Anopheles gambiae* and *Anopheles funestus* in Kenya. Malar J. 2009;8:268. 19941637 10.1186/1475-2875-8-268 PMC2793260

[12] Le PVV, Kumar P, Ruiz MO, Mbogo C, Muturi EJ. Predicting the direct and indirect impacts of climate change on malaria in coastal Kenya. PLoS ONE. 2019;14:e0211258. 30726279 10.1371/journal.pone.0211258 PMC6364917

[13] Martens WJ, Niessen LW, Rotmans J, Jetten TH, McMichael AJ. Potential impact of global climate change on malaria risk. Environ Health Perspect. 1995;103:458–64. 7656875 10.1289/ehp.95103458 PMC1523278

[14] Murdock CC, Sternberg ED, Thomas MB. Malaria transmission potential could be reduced with current and future climate change. Sci Rep. 2016;6:27771. 27324146 10.1038/srep27771 PMC4914975

[15] Mafwele BJ, Lee JW. Relationships between transmission of malaria in Africa and climate factors. Sci Rep. 2022;12:14392. 35999450 10.1038/s41598-022-18782-9 PMC9399114

[16] Koffi AA, Camara S, Ahoua Alou LP, Oumbouke WA, Wolie RZ, Tia IZ, et al. *Anopheles* vector distribution and malaria transmission dynamics in Gbêkê region, central Côte d’ivoire. Malar J. 2023;22:192. 37349819 10.1186/s12936-023-04623-1 PMC10288776

[17] Traoré B, Koutou O, Sangaré B. A mathematical model of malaria transmission dynamics with general incidence function and maturation delay in a periodic environment. Lett Biomath. 2020;7:37–54.

[18] DLP Madagascar. Plan stratégique National de lutte Contre Le paludisme 2018–2022 « elimination progressive du paludisme à Madagascar ». Madagascar: Ministère de la santé publique - Direction de Lutte contre le Paludisme; 2017.

[19] Abiodun GJ, Maharaj R, Witbooi P, Okosun KO. Modelling the influence of temperature and rainfall on the population dynamics of *Anopheles arabiensis*. Malar J. 2016;15:364. 27421769 10.1186/s12936-016-1411-6 PMC4946230

[20] White MT, Griffin JT, Churcher TS, Ferguson NM, Basáñez M-G, Ghani AC. Modelling the impact of vector control interventions on *Anopheles gambiae* population dynamics. Parasit Vectors. 2011;4:153. 21798055 10.1186/1756-3305-4-153 PMC3158753

[21] Depinay J-MO, Mbogo CM, Killeen G, Knols B, Beier J, Carlson J, et al. A simulation model of African Anopheles ecology and population dynamics for the analysis of malaria transmission. Malar J. 2004;3:29. 15285781 10.1186/1475-2875-3-29 PMC514565

[22] Cailly P, Tran A, Balenghien T, L’Ambert G, Toty C, Ezanno P. A climate-driven abundance model to assess mosquito control strategies. Ecol Model. 2012;227:7–17.

[23] Haramboure M, Labbé P, Baldet T, Damiens D, Gouagna LC, Bouyer J, et al. Modelling the control of *Aedes albopictus* mosquitoes based on sterile males release techniques in a tropical environment. Ecol Model. 2020;424:109002.

[24] Tran A, Fall AG, Biteye B, Ciss M, Gimonneau G, Castets M, et al. Spatial modeling of mosquito vectors for rift Valley fever virus in Northern senegal: integrating satellite-derived meteorological estimates in population dynamics models. Remote Sens. 2019;11:1024.

[25] Tran A, Mangeas M, Demarchi M, Roux E, Degenne P, Haramboure M, et al. Complementarity of empirical and process-based approaches to modelling mosquito population dynamics with *Aedes albopictus* as an example—application to the development of an operational mapping tool of vector populations. PLoS ONE. 2020;15:e0227407. 31951601 10.1371/journal.pone.0227407 PMC6968851

[26] Pachka H, Annelise T, Alan K, Power T, Patrick K, Véronique C, et al. Rift Valley fever vector diversity and impact of meteorological and environmental factors on culex pipiens dynamics in the Okavango delta. Botsw Parasites Vectors. 2016;9:434. 10.1186/s13071-016-1712-1 PMC4977755 27502246

[27] CREAM. Monographie région atsimo Atsinanana. Madagascar; 2013.

[28] Cailly P, Balenghien T, Ezanno P, Fontenille D, Toty C, Tran A. Role of the repartition of wetland breeding sites on the spatial distribution of Anopheles and Culex, human disease vectors in Southern France. Parasit Vectors. 2011;4:65. 21548912 10.1186/1756-3305-4-65 PMC3114004

[29] Ndiaye A, Niang EHA, Diène AN, Nourdine MA, Sarr PC, Konaté L, et al. Mapping the breeding sites of *Anopheles gambiae* s. l. in areas of residual malaria transmission in central western Senegal. PLoS ONE. 2020;15:e0236607. 10.1371/journal.pone.0236607 PMC7732347 33306671

[30] Youssefi F, Javad Valadan Zoej M, Ali Hanafi-Bojd A, Borahani Dariane A, Khaki M, Safdarinezhad A. Predicting the location of larval habitats of *Anopheles* mosquitoes using remote sensing and soil type data. Int J Appl Earth Obs Geoinf. 2022;108:102746.

[31] Shaman J, Spiegelman M, Cane M, Stieglitz M. A hydrologically driven model of swamp water mosquito population dynamics. Ecol Model. 2006;194:395–404.

[32] Degenne P, Lo Seen D, Parigot D, Forax R, Tran A, Ait Lahcen A, et al. Design of a domain specific language for modelling processes in landscapes. Ecol Model. 2009;220:3527–35.

[33] Arisco NJ, Rice BL, Tantely LM, Girod R, Emile GN, Randriamady HJ, et al. Variation in Anopheles distribution and predictors of malaria infection risk across regions of Madagascar. Malar J. 2020;19:348. 32993669 10.1186/s12936-020-03423-1 PMC7526177

[34] Goupeyou-Youmsi J, Rakotondranaivo T, Puchot N, Peterson I, Girod R, Vigan-Womas I, et al. Differential contribution of *Anopheles coustani* and *Anopheles arabiensis* to the transmission of *Plasmodium falciparum* and *Plasmodium vivax* in two neighbouring villages of Madagascar. Parasit Vectors. 2020;13:430. 32843082 10.1186/s13071-020-04282-0 PMC7447585

[35] Nepomichene TN, Tata E, Boyer S. Malaria case in Madagascar, probable implication of a new vector, Anopheles coustani. Malar J. 2015;14:475. 26620552 10.1186/s12936-015-1004-9 PMC4666205

[36] Ezanno P, Aubry-Kientz M, Arnoux S, Cailly P, L’Ambert G, Toty C, et al. A generic weather-driven model to predict mosquito population dynamics applied to species of Anopheles, culex and Aedes genera of Southern France. Prev Vet Med. 2015;120:39–50. 25623972 10.1016/j.prevetmed.2014.12.018

[37] Tompkins AM, Ermert V. A regional-scale, high resolution dynamical malaria model that accounts for population density, climate and surface hydrology. Malar J. 2013;12:65. 23419192 10.1186/1475-2875-12-65 PMC3656787

[38] Ngowo HS, Okumu FO, Hape EE, Mshani IH, Ferguson HM, Matthiopoulos J. Using bayesian state-space models to understand the population dynamics of the dominant malaria vector, *Anopheles funestus* in rural Tanzania. Malar J. 2022;21(1):161. 35658961 10.1186/s12936-022-04189-4 PMC9166306

[39] Taconet P, Porciani A, Soma D, Mouline K, Simard F, Koffi A, et al. Data-driven and interpretable machine-learning modeling to explore the fine-scale environmental determinants of malaria vectors biting rates in rural Burkina Faso. Parasit Vectors. 2021;14:345. 10.1186/s13071-021-04851-x PMC8243492 34187546

[40] Nepomichene TN, Andrianaivolambo L, Boyer S, Bourgouin C. Efficient method for establishing F1 progeny from wild populations of Anopheles mosquitoes. Malar J. 2017;16:21. 10.1186/s12936-017-1681-7 PMC5223328 28069024

[41] Chobu M, Nkwengulila G, Mahande AM, Mwang’onde BJ, Kweka EJ. Direct and indirect effect of predators on *Anopheles gambiae* sensu stricto. Acta Trop. 2015;142:131–7. 25438260 10.1016/j.actatropica.2014.11.012

[42] Kweka EJ, Zhou G, Beilhe LB, Dixit A, Afrane Y, Gilbreath TM, et al. Effects of co-habitation between *Anopheles gambiae* s.s. and *culex quinquefasciatus* aquatic stages on life history traits. Parasit Vectors. 2012;5:33. 22321562 10.1186/1756-3305-5-33 PMC3293084

[43] Kweka EJ, Tenu F, Magogo F, Mboera LEG. *Anopheles gambiae* sensu stricto aquatic stages development comparison between insectary and semifield structure. Adv Zool. 2015;2015:1–6.

[44] Lyons CL, Coetzee M, Chown SL. Stable and fluctuating temperature effects on the development rate and survival of two malaria vectors, *Anopheles arabiensis* and *Anopheles funestus*. Parasit Vectors. 2013;6:104. 23590860 10.1186/1756-3305-6-104 PMC3637585

[45] Manda H, Gouagna LC, Foster WA, Jackson RR, Beier JC, Githure JI, et al. Effect of discriminative plant-sugar feeding on the survival and fecundity of *Anopheles gambiae*. Malar J. 2007;6:113. 17711580 10.1186/1475-2875-6-113 PMC2034389

[46] Olayemi IK, Ande AT. Life table analysis of Anopheles Gambiae (Diptera: Culicidae) in relation to malaria transmission. J VECTOR BORNE DIS. 2009;4:295-8 . 19959856

[47] Tchigossou GM, Akoton R, Yessoufou A, Djegbe I, Zeukeng F, Atoyebi SM, et al. Water source most suitable for rearing a sensitive malaria vector, Anopheles funestus in the laboratory [version 2; referees: 2 approved]. Wellcome Open Res. 2018;2:109. 10.12688/wellcomeopenres.12942.2 PMC5721565 29387806

[48] Jetten TH, Takken W. Anophelism without malaria in europe: a review of the ecology and distribution of the genus Anopheles in Europe. Wageningen, The Netherlands: Wageningen Agricultural University; 1994.

[49] Maharaj R. Life table characteristics of Anopheles arabiensis (Diptera: Culicidae) under simulated seasonal conditions. Me. 2003;40:737–42. 10.1603/0022-2585-40.6.737 14765646

