# Correction: Spatial modeling of the population dynamics of *Anopheles* mosquitoes in Madagascar

**DOI:** 10.1186/s12942-025-00443-5

**Published:** 2026-04-30

**Authors:** Hobiniaina Anthonio Rakotoarison, Thiery Nirina Nepomichene, Hélène Guis, Romain Girod, Solofoarisoa Rakotoniaina, Fanjasoa Rakotomanana, Annelise Tran

**Affiliations:** 1 Epidemiology and Clinical Research Unit, Institut Pasteur de Madagascar, Antananarivo, Madagascar; 2 CIRAD, UMR TETIS, Montpellier, France; 3 TETIS, Univ Montpellier, AgroParisTech, CIRAD, CNRS, INRAE, Montpellier, France; 4 LGET, IOGA, Université d’Antananarivo, Antananarivo, Madagascar; 5 Medical Entomology Unit, Institut Pasteur de Madagascar, Antananarivo, Madagascar; 6 CIRAD, UMR ASTRE, Antananarivo, Madagascar; 7 ASTRE, Univ Montpellier, CIRAD, INRAE, Montpellier, France; 8 Epidemiology and Public Health Unit, Institut Pasteur du Cambodge, Phnom Penh, Cambodia; 9 CIRAD, UMR ASTRE, Montpellier, France

**Correction: International Journal of Health Geographics (2025) 24:34**

10.1186/s12942-025-00424-8

In this article [1], the author would like to correct the few typographical errors in the sections “Description of the model”, “Simulation”, “Validation” and also like to update the acknowledgements as well. The incorrect and corrected text are provided in this correction.

The first sentence of the second paragraph in the section **Description of the model** should be “First, the eggs (*E*) become larvae (*L*) which then become pupae (*P*). After emergence, the emerging females (*Aem*) are considered nulliparous (i.e., females that have never laid eggs) in search of a host (*A1ℎ*), i.e. in search of a blood meal, then engorged (*A1g*), and then in search for an oviposition site (*A1o*). After having laid eggs once, they become parous females in search of a host (*A2ℎ*), then engorged (*A2g*) and finally in search for an oviposition site (*A2o*) (Fig. 2).” instead of “First, the eggs () become larvae () which then become pupae (). After emergence, the emerging females () are considered nulliparous (i.e., females that have never laid eggs) in search of a host (1 h), i.e., in search of a blood meal, then engorged (1), and then in search for an oviposition site (1). After having laid eggs once, they become parous females in search of a host (2 h), then engorged (2) and finally in search for an oviposition site (2) (Fig. 2).”

The last sentence of the first paragraph in the Section **Simulation** should be “… of the number of individuals in each of the *Anopheles* developmental stages (*E, L, P, Aem, A1h, A1g, A1o, A2h, A2g, A2o*) in each commune of the three districts considered.” Instead of “… of the number of individuals in each of the Anopheles developmental stages (,, ,, 1 h, 1, 1, 2 h, 2, 2) in each commune of the three districts considered.”

The first sentence of the first paragraph in the section **Validation** should be “The model was validated by comparing the predicted host-seeking density of *Anopheles* (*Aℎ = A1ℎ + A2ℎ*) with entomological data provided by the Medical Entomology Unit of the Institut Pasteur of Madagascar.” instead of “The model was validated by comparing the predicted host-seeking density of Anopheles (h = 1 h + 2 h) with entomological data provided by the Medical Entomology Unit of the Institut Pasteur of Madagascar.”

Finally, in the section “Results / Temporal dynamics”, title of Fig. 3 should be “Population dynamics of host-seeking *Anopheles* at the three sites in Farafangana District. *The curves correspond to predicted densities*,* the dots represent field observations*,* and the pie-charts represent the breeding sites*” instead of “Population dynamics of host-seeking Anopheles at the three sites in Farafangana District. *The curves correspond to predicted densities*,* the dots represent field observations*,* and the camemberst represent the breeding sites*”.

In addition, the author would like to amend the acknowledgements as below:

The authors would like to acknowledge Luciano Tantely for his valuable advice and expertise in entomology. The authors also thank the platform in partnership One Health Indian Ocean (www.onehealth-oi.org), the ANISETTE project, CIRAD through the AI Doctorant program, and the French Embassy through the French Government Scholarship for their support.

The original article has been corrected.
