# Supplementary material for: Spatial modeling of the population dynamics of Anopheles mosquitoes in Madagascar
Source: Int J Health Geogr. 2025 Nov 18;24:34. doi: 10.1186/s12942-025-00424-8 (PMC12625280; doi:10.1186/s12942-025-00424-8)
Supplement: Supplementary file 1 — Supplementary Material 1 [file 12942_2025_424_MOESM1_ESM.docx]

**Additional file 1**

**Equation S1.** Differential equation system of the model

$$\left\{ \begin{matrix} \dot{E}=\gamma_{Ao}\left( \beta_{1}A_{1o} \right.+\beta_{1}A_{1o})-\left( \mu_{E}+f_{E} \right)E \\ \begin{matrix} \dot{L}=f_{E}E-\left[ m_{L}\left( 1+\frac{L}{k_{L}} \right)+f_{L} \right]L \\ \dot{P}=f_{L}L-\left[ m_{P}+f_{P} \right]P \\ \dot{A_{em}}=f_{P}P\sigma e^{-\mu_{em}\left( 1+\frac{P}{k_{P}} \right)}-\left[ m_{A}+\gamma_{A_{em}} \right]A_{em} \end{matrix} \\ \begin{matrix} \dot{A_{1h}}=\gamma_{A_{em}}A_{em}-\left( m_{A}+\mu_{r}+\gamma_{Ah} \right)A_{1h} \\ \begin{matrix} \dot{A_{1g}}=\gamma_{Ah}A_{1h}-\left( m_{A}+f_{Ag} \right)A_{1g} \\ \begin{matrix} \dot{A_{1o}}=f_{Ag}A_{1g}-\left( m_{A}+\mu_{r}+\gamma_{Ao} \right)A_{1o} \\ \begin{matrix} \dot{A_{2h}}=\gamma_{Ao}\left( A_{1o}+A_{2o} \right)-\left( m_{A}+\mu_{r}+\gamma_{Ah} \right)A_{2h} \\ \dot{A_{2g}}=\gamma_{Ah}A_{2h}-\left( m_{A}+f_{Ag} \right)A_{2g} \\ \dot{A_{2o}}=f_{Ag}A_{2g}-\left( m_{A}+\mu_{r}+\gamma_{Ao} \right)A_{2o} \end{matrix} \end{matrix} \end{matrix} \end{matrix} \end{matrix} \right.$$

Model parameters are represented by Greek letters and are constant while model functions, indicated by Latin letters, are climate-driven.

For each development stage X, $\gamma_{X}$ indicates the transition rate and $f_{X}$ the transition function to the next stage, $\mu_{X}$ denotes the mortality rate and $m_{X}$ the mortality function, $\beta_{X}$ refers to egg-laying rate and $k_{X}$ represents the environmental carrying capacity.

The sex ratio at emergence, $\sigma$, solely considers the proportion of emerging females that reach the adult stage.
