# Supplementary material for: Spatial modeling of the population dynamics of Anopheles mosquitoes in Madagascar
Source: Int J Health Geogr. 2025 Nov 18;24:34. doi: 10.1186/s12942-025-00424-8 (PMC12625280; doi:10.1186/s12942-025-00424-8)
Supplement: Supplementary file 2 — Supplementary Material 2 [file 12942_2025_424_MOESM2_ESM.docx]

**Additional file 2.**

**Figures A1 – A7.** Figures comparing simulations with and without the agricultural calendar across study sites and *Anopheles* species


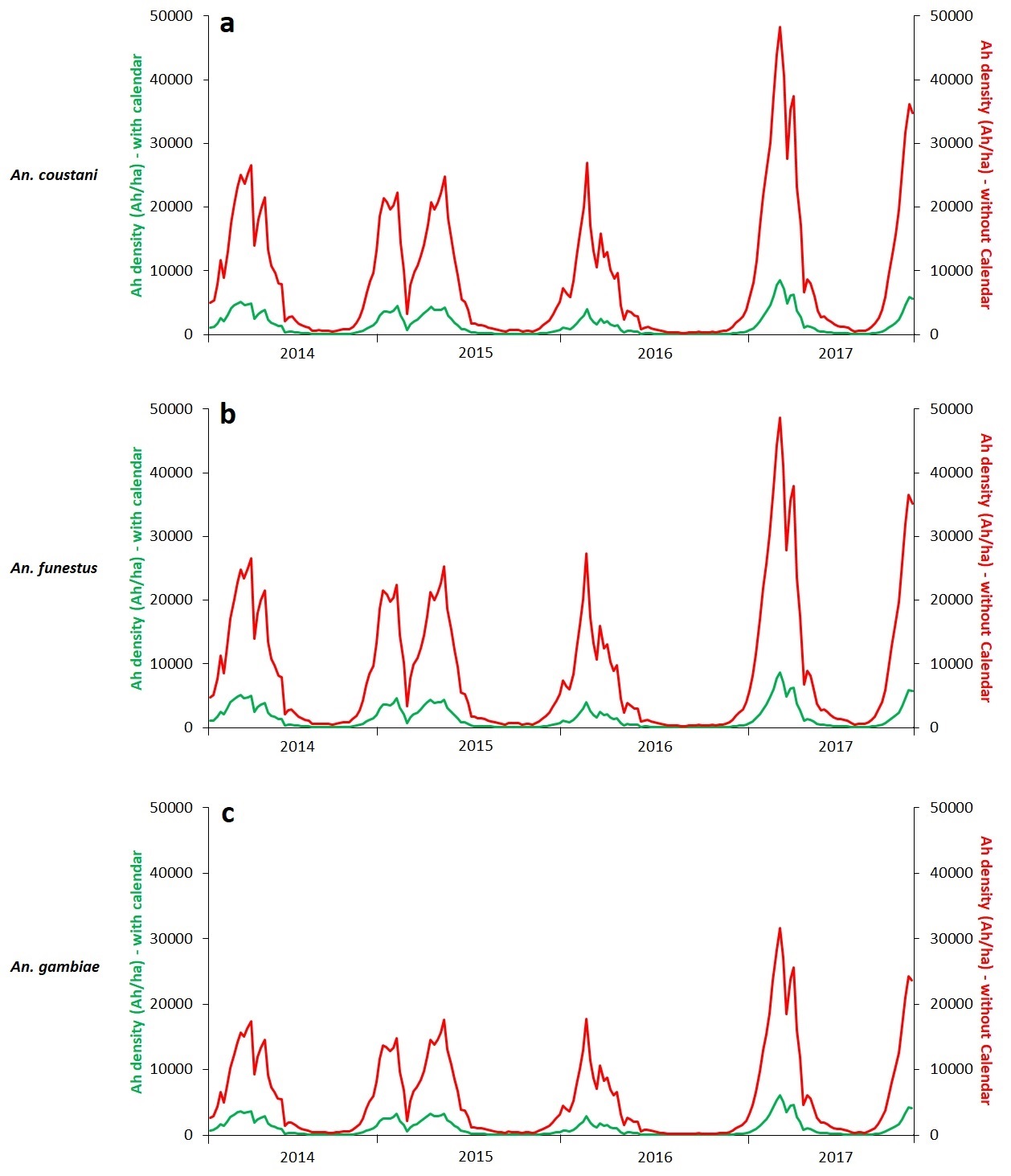


Figure A1. Comparison of simulations with and without the agricultural calendar in Ambahibe (Farafangana). a: An. coustani, b: An. funestus, c: An. gambiae


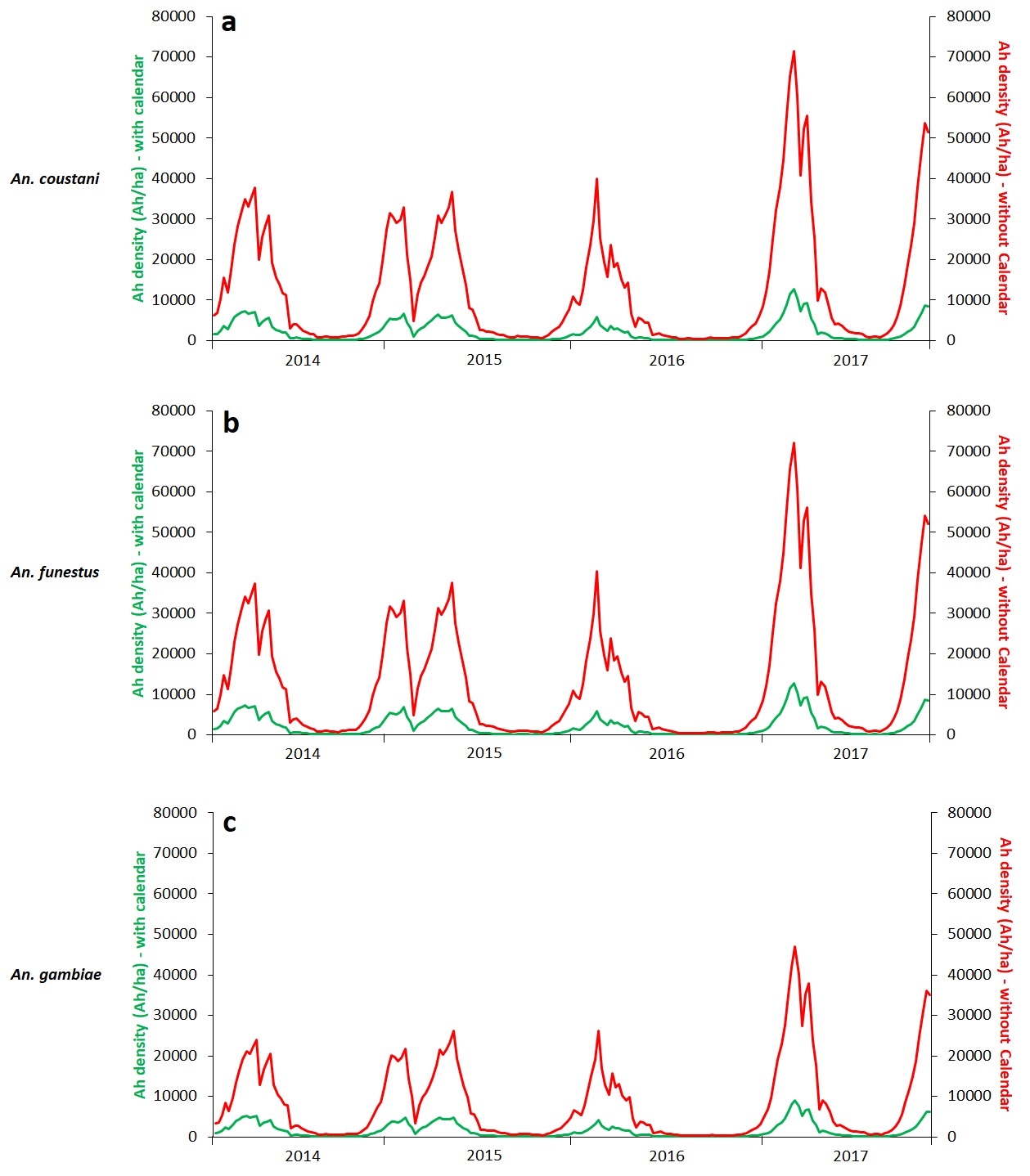


Figure A2. Comparison of simulations with and without the agricultural calendar in Mahasoa (Farafangana). a: An. coustani, b: An. funestus, c: An. gambiae


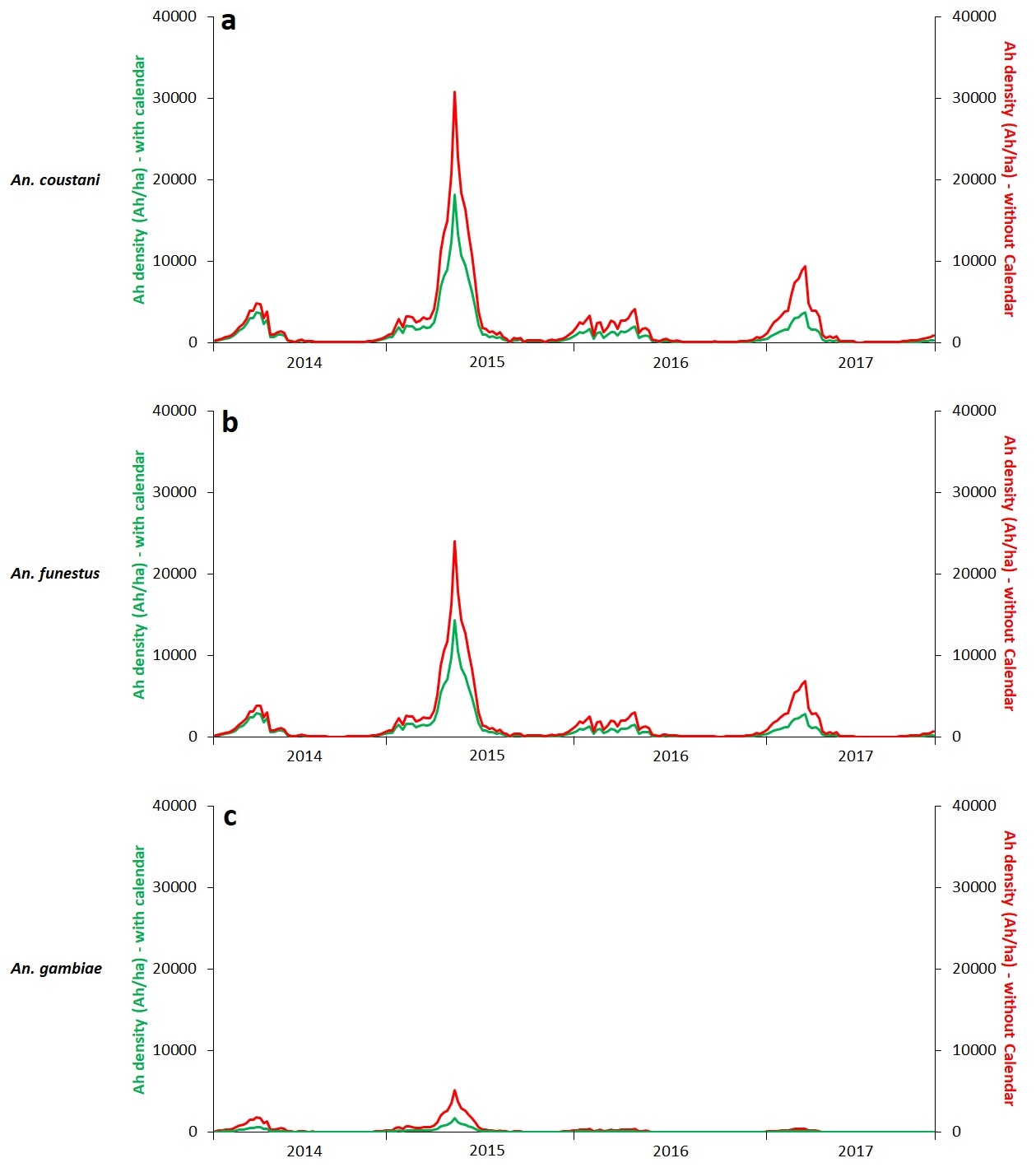


Figure A3. Comparison of simulations with and without the agricultural calendar in Vohimasy (Farafangana). a: An. coustani, b: An. funestus, c: An. gambiae


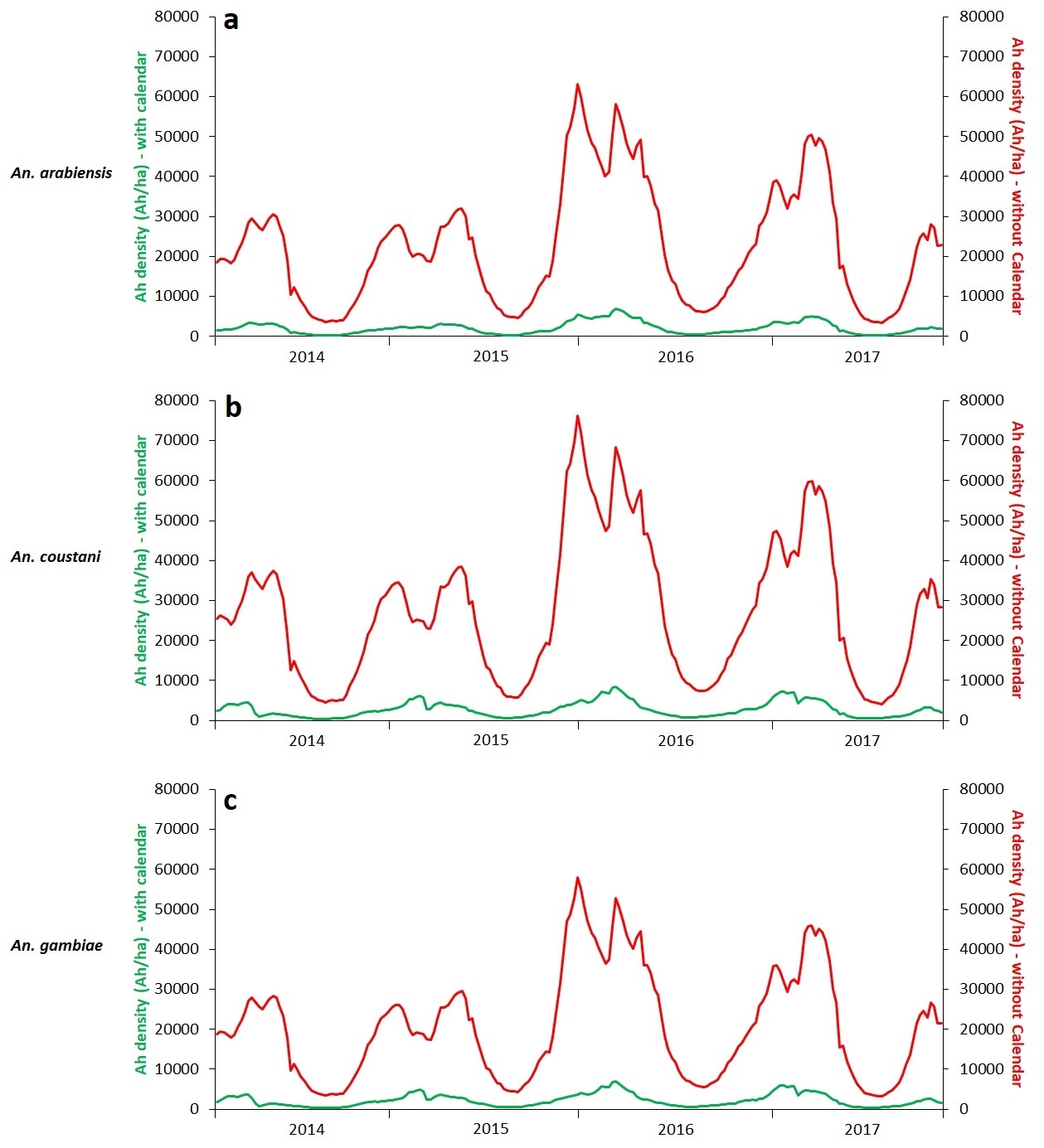


Figure A4. Comparison of simulations with and without the agricultural calendar in Anosikely Avaratra (Maevatanana). a: An. arabiensis, b: An. coustani, c: An. gambiae


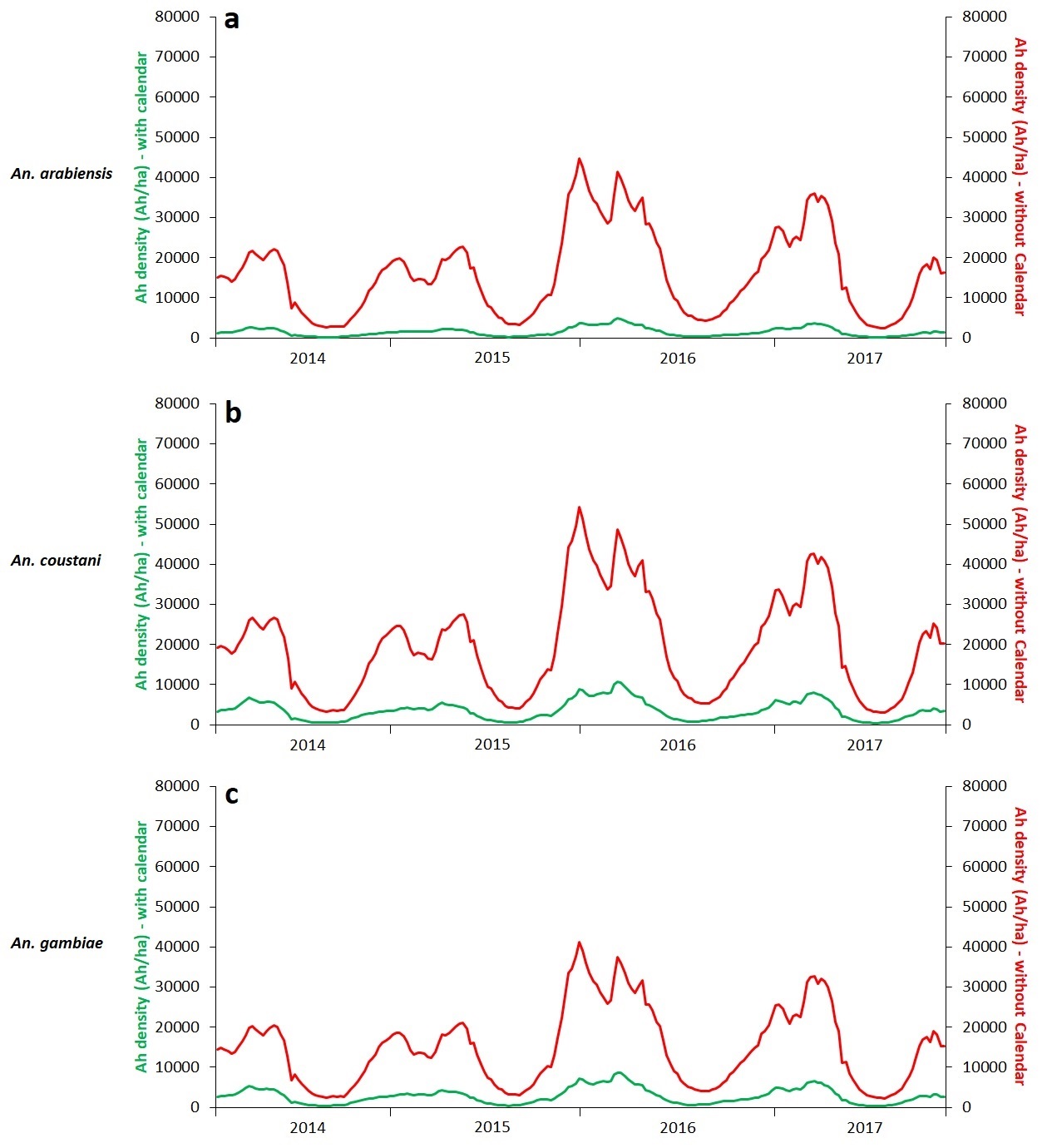


Figure A5. Comparison of simulations with and without the agricultural calendar in Morarano (Maevatanana). a: An. arabiensis, b: An. coustani, c: An. gambiae


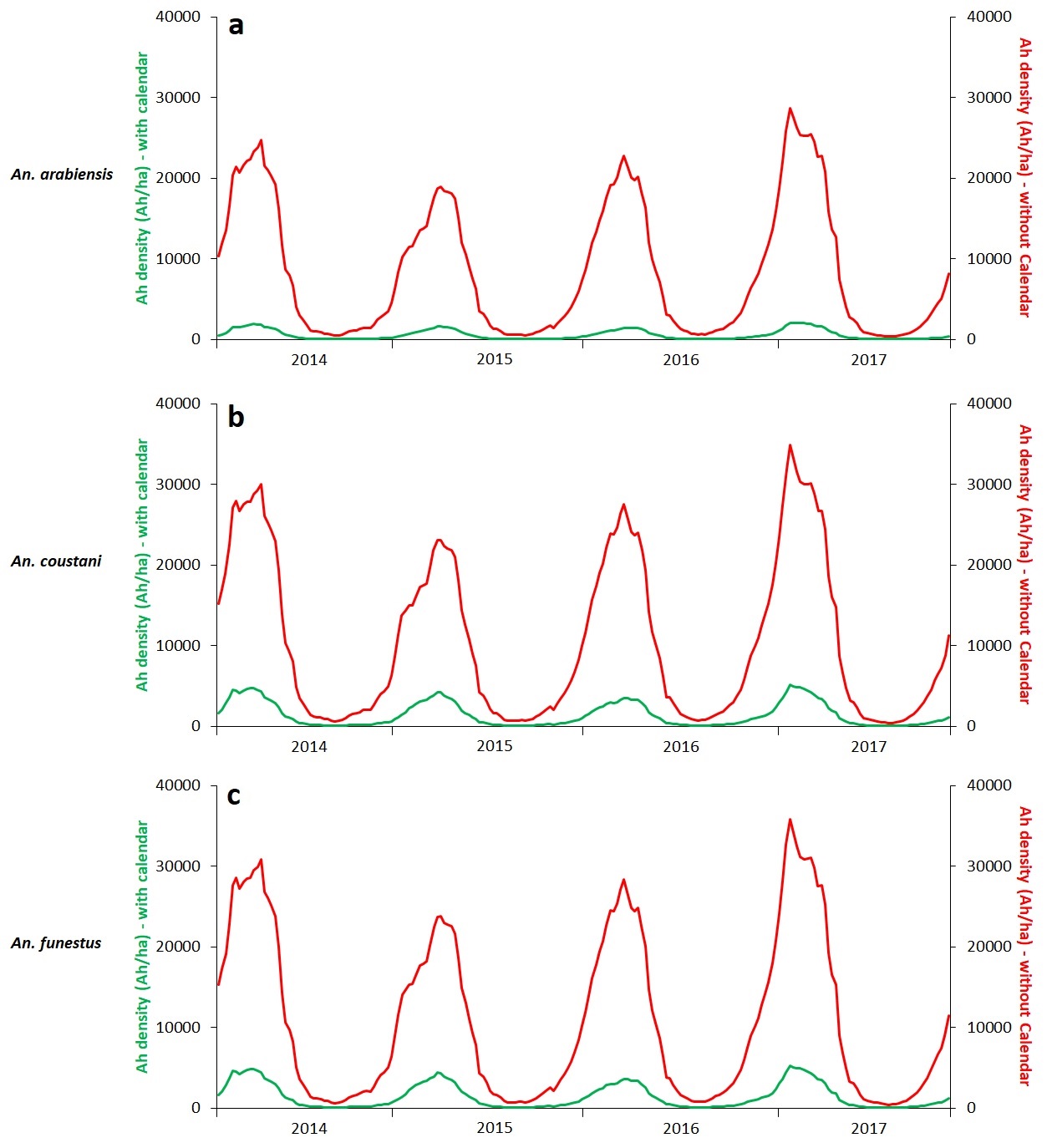


Figure A6. Comparison of simulations with and without the agricultural calendar in Ampasy (Morondava). a: An. arabiensis, b: An. coustani, c: An. funestus


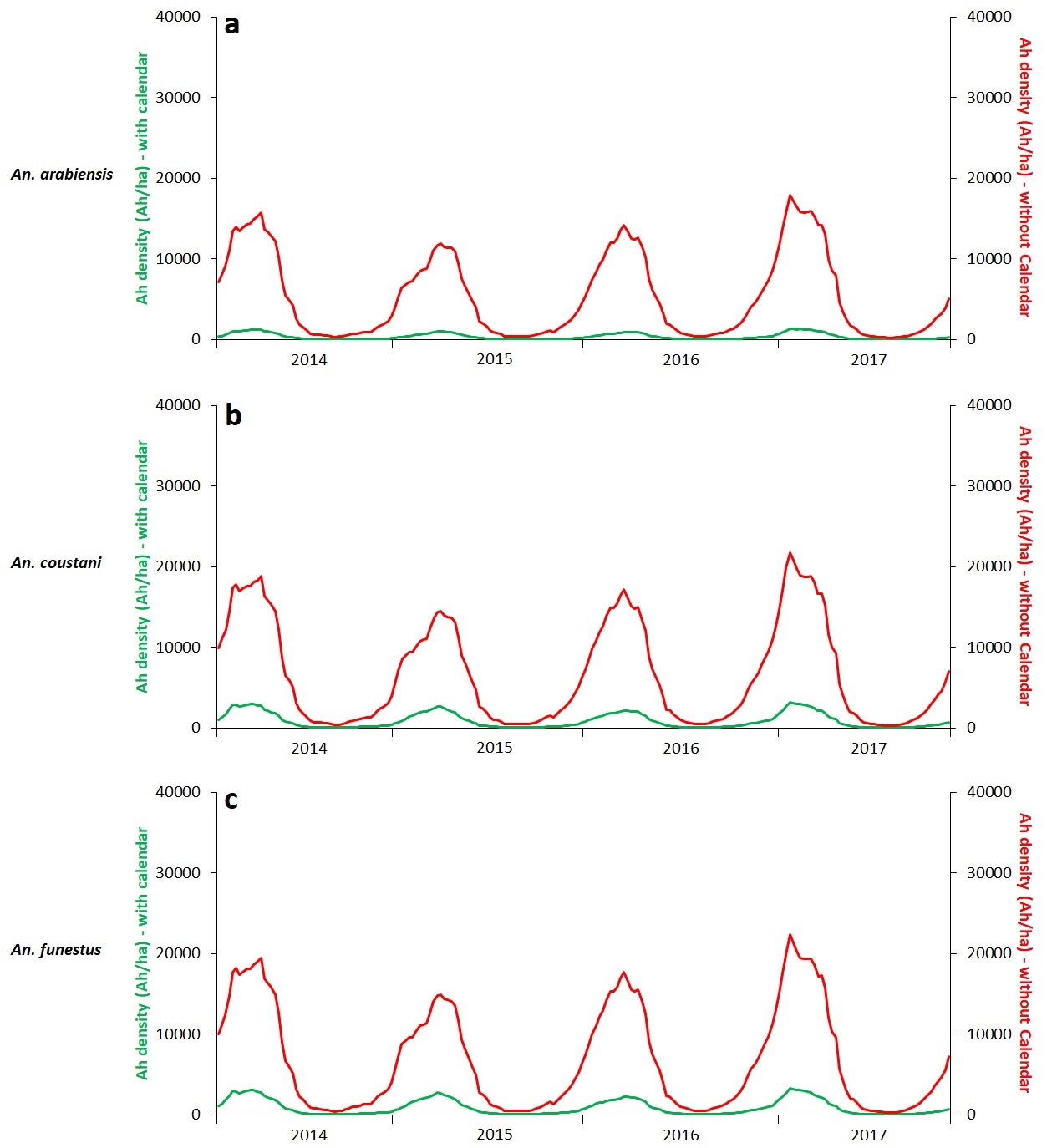


Figure A7. Comparison of simulations with and without the agricultural calendar in Antsakoameloka (Morondava). a: An. arabiensis, b: An. coustani, c: An. funestus
